# Integrative proteomics highlight presynaptic alterations and c-Jun misactivation as convergent pathomechanisms in ALS

**DOI:** 10.1007/s00401-023-02611-y

**Published:** 2023-07-24

**Authors:** Amr Aly, Zsofia I. Laszlo, Sandeep Rajkumar, Tugba Demir, Nicole Hindley, Douglas J. Lamont, Johannes Lehmann, Mira Seidel, Daniel Sommer, Mirita Franz-Wachtel, Francesca Barletta, Simon Heumos, Stefan Czemmel, Edor Kabashi, Albert Ludolph, Tobias M. Boeckers, Christopher M. Henstridge, Alberto Catanese

**Affiliations:** 1 Institute of Anatomy and Cell Biology, Ulm University, Ulm, Germany; 2 Division of Cellular and Systems Medicine, School of Medicine, University of Dundee, Dundee, Scotland, UK; 3 FingerPrints Proteomics Facility, Discovery Centre, School of Life Sciences, University of Dundee, Dundee, Scotland, UK; 4 Proteome Center Tübingen, University of Tübingen, 72076 Tübingen, Germany; 5 Quantitative Biology Center (QBiC), University of Tübingen, 72076 Tübingen, Germany; 6 Biomedical Data Science, Department of Computer Science, University of Tübingen, 72076 Tübingen, Germany; 7 Laboratory of Translational Research for Neurological Disorders, Imagine Institute, Université de Paris, INSERM, UMR 1163, 75015 Paris, France; 8 Department of Neurology, Ulm University School of Medicine, Ulm, Germany; 9 German Center for Neurodegenerative Diseases (DZNE), Ulm Site, Germany

**Keywords:** ALS, Spinal cord, Proteomics, Synapse, hiPSC, Motoneuron

## Abstract

Amyotrophic Lateral Sclerosis (ALS) is a fatal neurodegenerative disease mainly affecting upper and lower motoneurons. Several functionally heterogeneous genes have been associated with the familial form of this disorder (fALS), depicting an extremely complex pathogenic landscape. This heterogeneity has limited the identification of an effective therapy, and this bleak prognosis will only improve with a greater understanding of convergent disease mechanisms. Recent evidence from human *post-mortem* material and diverse model systems has highlighted the synapse as a crucial structure actively involved in disease progression, suggesting that synaptic aberrations might represent a shared pathological feature across the ALS spectrum. To test this hypothesis, we performed the first comprehensive analysis of the synaptic proteome from *post-mortem* spinal cord and human iPSC-derived motoneurons carrying mutations in the major ALS genes. This integrated approach highlighted perturbations in the molecular machinery controlling vesicle release as a shared pathomechanism in ALS. Mechanistically, phosphoproteomic analysis linked the presynaptic vesicular phenotype to an accumulation of cytotoxic protein aggregates and to the pro-apoptotic activation of the transcription factor c-Jun, providing detailed insights into the shared pathobiochemistry in ALS. Notably, sub-chronic treatment of our iPSC-derived motoneurons with the fatty acid docosahexaenoic acid exerted a neuroprotective effect by efficiently rescuing the alterations revealed by our multidisciplinary approach. Together, this study provides strong evidence for the central and convergent role played by the synaptic microenvironment within the ALS spinal cord and highlights a potential therapeutic target that counteracts degeneration in a heterogeneous cohort of human motoneuron cultures.

**Supplementary Information:**

The online version contains supplementary material available at 10.1007/s00401-023-02611-y.

## Introduction

Amyotrophic lateral sclerosis (ALS), the most common motoneuron (MN) disease, is characterized by the loss of upper and lower MNs leading to an invariably fatal outcome generally within 1–5 years from symptoms´ onset [63]. The genetic component of ALS is well recognized with approximately 10% of patients displaying Mendelian inherited disease with high penetrance (familial ALS—fALS) [77]. The identification of the most prevalent genes involved in ALS like *C9ORF72, TARDBP, SOD1* and *FUS* has revealed an extreme mechanistic heterogeneity. The genetic causes of ALS are linked to genes involved in several different cellular pathways such as DNA-damage repair, protein degradation and RNA processing [46]. This has represented a major obstacle in the identification of therapeutic strategies that can be applied across the broad spectrum of ALS patients. Indeed, the only drugs approved to treat this disease, riluzole and edaravone, are only mildly beneficial in a subset of individuals [20]. Thus, despite extensive efforts trying to dissect the specific molecular pathomechanisms underlying MN loss in ALS, the dynamics driving disease progression are poorly understood. To develop a drug that benefits all ALS patients a greater understanding of convergent disease mechanisms is needed. One common feature described in human *post-mortem* material and across the majority of heterogeneous animal models of ALS is the early, pre-symptomatic loss of synapses [28, 38]. Therefore, the specific contribution of the synaptic microenvironment to the neurodegenerative processes, as well as the electrophysiological alterations characterizing the vulnerable neurons in ALS, have to be dissected. Despite several studies showing that synapses of ALS-related neurons undergo dramatic pathological alterations [28, 38], the question of whether these are the consequence or the cause of the degenerative processes remains unanswered. Evidence from both animal models and ALS patients showed that neurophysiological alterations of MN occur long before significant cellular degeneration, thus representing one of the earliest pathogenic features in ALS [45, 49]. Indeed, clinical neurophysiological studies have shown that presymptomatic mutation carriers exhibit altered excitability in the disease-vulnerable areas before the onset of symptoms [80], which include muscle twitching, cramps and fasciculations, as well as evidence of motor unit hyperactivation [5]. Of note, the pathological implications of this hyperexcitability are not clear, as recent works suggested that it might represent a compensatory mechanism set in motion by disease-resistant cells aimed at compensating the early loss of fast-fatigable (FF) motor units, which in contrast appear to be hypoexcitable [40, 45]. Despite the puzzling electrophysiological properties characterizing the vulnerable ALS-associated neurons [19, 34, 70, 74, 81], evidence highlights the synapse as a crucially important structure across the ALS spectrum. For example, synapse loss has been observed in C9orf72- and VCP-mutant MNs [14, 27] as well as in the spinal cord of the SOD1G93A mouse model [4]. Interestingly, the reduced number of synaptic contacts appears to be linked to impaired protein homeostasis within this microenvironment, as the accumulation of aberrant clusters of scaffolds and receptors have been detected in mutant MNs [14, 18, 70], as well as upon overexpression of mutant TDP-43 [58]. In addition, synaptic localization and function have been described for the protein product of some major ALS genes such as *C9orf72*, *FUS* and *TARDBP* [38, 66, 84, 86], suggesting that pathogenic mutations may also have a direct impact on synaptic physiology [53].

Taken together, the evidence indicates that molecular and structural alterations within the synaptic compartment dramatically impact MN functionality and might actively contribute to the selective loss of this vulnerable cellular population. In this study we performed an extensive analysis of the human spinal cord synaptic proteome in ALS, combining the powerful insight of *post-mortem* samples with hiPSC-derived neuronal cultures obtained from patients carrying pathogenic mutations in the most prevalent genes linked to the disease. Our aims were to highlight the exact synaptic aberrations underlying the electrophysiological abnormalities observed across the heterogeneous ALS spectrum and to identify novel therapeutic targets within this crucial neuronal structure. Utilizing this integrated approach, we discovered alterations in the presynaptic vesicular machinery that were convergent across all our ALS cultures and human *post-mortem* material. Importantly, we discovered a mechanistic link between protein aggregates, transcription factor phosphorylation and synapse loss, which were all rescued following treatment with the neuroprotective lipid docosahexaenoic acid (DHA). This study significantly improves our understanding of convergent synaptic changes in ALS and highlights a potential mechanism for therapeutic interrogation.

## Material and methods

### Donor characteristics and human brain collection

Cervical spinal cord tissue was extracted from all donors. Controls (*n* = 10) and ALS patients were stratified according to disease familial history and by the presence of *C9orf72* repeat expansion (*n* = 10) and TDP-43 pathology (*n* = 10). The summary of donor information can be found in Table 1 and individual donor details in Supplementary Table 1.

**Table 1:** Summary demographic information of donors. Data presented as mean ± SD

Experimental group	*n*	Age at death (years)	*Post-mortem* intervals (hrs)	Gender (% Male)
Control	10	60 ± 11	90 ± 29	50
Sporadic ALS with TDP-43 pathology	10	67 ± 13	72 ± 20	60
*C9orf72* repeat expansion -positive	10	62 ± 10	55 ± 29	50

### Preparation of synaptoneurosomes from human *post-mortem* tissue

*Post-mortem* human cervical spinal cord tissue was homogenized, filtered and centrifuged to yield synaptoneurosome preparations as described [38]. In summary, spinal cord tissue was homogenized using glass homogenizers on ice with homogenization buffer (25 mM HEPES (pH 7.5), 120 mM NaCl, 5 mM KCl, 1 mM MgCl_2_, 2 mM CaCl_2_) with the addition of protease and phosphatase inhibitors (Roche #11,836,153,001, Thermo Scientific #A32959). The homogenized tissue was filtered using 80 µm nylon filter, resulting in a total homogenate sample (TH), which was partially aliquoted and stored on dry ice. A 5 µm filter (Millipore, SLSV025LS) was then used to filter the remainder of the TH and the sample underwent a 5 min centrifugation at 1000 × g. The pellets generated went through a series of washes in homogenization buffer and disposal of supernatant before the final pellet was weighed and resuspended in protein extraction buffer (100 mM Tris–HCL Buffer (pH 7.6) 4% SDS with 1% protease inhibitor cocktail (Roche #11,836,153,001)) in a 1:5 dilution in relation to pellet weight. Further sample homogenisation by hand occurred before final centrifugation at 17,000 × g at 4 °C for 20 min, from which the pellet was disposed of and the supernatant collected as the extracted synaptoneurosome (SNS) proteome. Micro BCA Protein Assay Kit (Thermo Scientific #23,235) was then used to determine the protein concentration of the samples for further experiments.

Extracted SNS protein (25 µg) from each individual case was pooled based on stratification of the presence of C*9orf72* repeat expansion, TDP-43 pathology and control. This pooling resulted in 3 representative groups, each consisting of 10 individual cases, for proteomic analysis (pool 1 – control (*n* = 10), pool 2—ALS-*C9orf72* (*n* = 10), pool 3–sporadic ALS with TDP-43 pathology (*n* = 10)). The remaining SNS sample from each individual was kept for any further validation experiments.

### Human iPSCs

The hiPSC lines used in this study are listed in Table 2. HiPSCs were cultured at 37 °C (5% CO_2_, 5% O_2_) on Matrigel^®^-coated (Corning, 354,277) 6-well plates using mTeSR1 medium (Stem Cell Technologies, 83,850). At 80% confluence, the colonies were detached using Dispase (Stem Cell Technologies, 07923) and passaged in a 1:3 or 1:6 split ratio. Analysis of mycoplasma contamination was performed using the MycoStrip™—Mycoplasma Detection Kit (Invivogen, rep-mysnc-50).

**Table 2:** Detailed information on the hiPSC lines used in this study

Group	ID	Gene	Mutation	Age	Sex	Origin	Catalog #
ALS	ALS^C9orf72^ I	C9orf72	(G_4_C_2_)_1.8 kb_	60	♂	Ulm University	NA
	ALS^C9orf72^ II	C9orf72	(G_4_C_2_)_6-8 kb_	46	♂	Cedars-Sinai	CS29iALS-C9
	ALS^C9orf72^ III	C9orf72	(G_4_C_2_)_2.7 kb_	50	♀	Cedars-Sinai	CS30iALS-C9
	ALS^FUS^ I	FUS	p.R521C	57	♀	Ulm University	NA
	ALS^FUS^ II	FUS	c.1484delG	27	♂	Ulm University	NA
	ALS^FUS^ III	FUS	c.1504delG	19	♂	Ulm University	NA
	ALS^TARDBP^ I	TARDBP	p.Gly298Ser	62	♂	Cedars-Sinai	CS47iALS-TDP
	ALS^TARDBP^ II	TARDBP	p.N390D	26	♂	Cedars-Sinai	CS5ZLDiALS
	ALS^SOD1^ I	SOD1	p.A5V	40	♀	Cedars-Sinai	CS07iALS-SOD1A4
	ALS^SOD1^ II	SOD1	p.G94A	57	♂	Cedars-Sinai	CS2RJViALS
	ALS^TBK1^ I	TBK1	p.Thr77TrpfsX4	41	♂	Ulm University	NA
	ALS^TBK1−FUS^	TBK1 FUS	p.Tyr185X p.R524G	54	♀	Ulm University	NA
Control	Healthy I	NA	NA	45	♀	Ulm University	NA
	Healthy II	NA	NA	64	♂	BioCat GmbH	SC600A-WT
	Healthy III	NA	NA	49	♂	Cedars-Sinai	CS0YX7iCTR
	Healthy IV	NA	NA	52	♀	Cedars-Sinai	CS14iCTR-21
	Corrected^C9orf72^	C9orf72	CRISPR-corrected (G_4_C_2_)_6-8 kb_	46	♂	Cedars-Sinai	CS29iALS-C9n1.ISO

### Differentiation of hiPSC-derived motoneurons

Motoneuron differentiation was carried out as previously described [14]. Briefly, hiPSC colonies were detached and cultivated in suspension in ultra-low attachment flasks T75 for 3 days for the formation of embryoid bodies (EBs) in hESC medium (DMEM/F12 + 20% knockout serum replacement + 1% NEAA + 1% β-mercaptoethanol + 1% antibiotic–antimycotic + SB-431542 10 µM + Dorsomorphin 1 µM + CHIR 99021 3 µM + Purmorphamine 1 µM + Ascorbic Acid 200 ng/µL + 1% B27 + 0.5% N2). On the fourth day, the medium was switched to MN Medium (DMEM/F12 + 24 nM sodium selenite + 16 nM progesterone + 0.08 mg/mL apotransferrin + 0.02 mg/mL insulin + 7.72 μg/mL putrescine + 1% NEAA, 1% antibiotic–antimycotic + 50 mg/mL heparin + 10 μg/mL of the neurotrophic factors BDNF, GDNF, and IGF-1, SB-431542 10 µM, Dorsomorphin 1 µM, CHIR 99021 3 µM, Purmorphamine 1 µM, Ascorbic Acid 200 ng/µL, Retinoic Acid 1 µM, cAMP 1 µM, 1% B27, 0.5% N2). Ultimately, after 5 further days of cultivation EBs were dissociated into single cells with Accutase (Sigma Aldrich) and plated onto μDishes, 24-well μPlates (Ibidi) or 6-well plates (Corning) pre-coated with Growth Factor Reduced Matrigel (Corning).

### Synaptosome isolation from hiPSC-derived motor neurons

Synaptosomes were isolated from DIV42 hiPSC-derived motor neurons (MNs) as previously described [59]. hiPSC-MNs, plated on 6-well tissue culture plates at a seeding density of 300,000 cells/well, were washed quickly with DPBS twice, and gently scraped and collected in 1 ml of Buffer A (10 mM HEPES pH 7.4, 2 mM EDTA, 5 mM sodium orthovanadate, 30 mM sodium fluoride, 20 mM β-glycerol phosphate, protease inhibitor cocktail). The cells were homogenized for 30 strokes using a glass Dounce tissue grinder (Kimble, 885,302–0002 and 885,303–0002) and the homogenate (Ho) was centrifuged at 500 × *g* for 5 min at 4 °C. The supernatant containing the nuclei- and cell debris-free total lysate (S1) was further centrifuged at 10,000 × *g* for 15 min at 4 °C to yield a cytosolic supernatant (S2) and the crude synaptosomal pellet (P2). Synaptosomes were obtained by resuspending P2 in 200 µl Buffer B (10 mM HEPES pH 7.4, 2 mM EDTA, 2 mM EGTA, 5 mM sodium orthovanadate, 30 mM sodium fluoride, 20 mM β-glycerol phosphate, 1% TritonX, protease inhibitor cocktail). Further centrifugation at 20,000 × *g* for 80 min at 4 °C led to the separation of the soluble, synaptic cytosol (S3) fraction in the supernatant and the insoluble, enriched postsynaptic density (P3) fraction as the pellet. The P3 fraction was then resuspended in 70 µl of Buffer C (50 mM Tris pH 9.0, 5 mM sodium orthovanadate, 30 mM sodium fluoride, 20 mM β-glycerol phosphate, 1% sodium deoxycholate, protease inhibitor cocktail). Protein concentration was estimated using the BCA method according to the manufacturer’s instructions (ThermoFisher Scientific, 23,227). Finally, 20 µg each of the S1 and P2 fractions were flash-frozen in liquid nitrogen and shipped with dry ice for proteomic analysis. S1, S3 and P3 fractions were used for Western blot experiments.

### Liquid chromatography–mass spectrometry

100 µg of total protein per group, obtained from *post-mortem* spinal cord samples, was processed using S-trap mini protocol (Protifi) as described [38]. Processing of 3 sample repeats yielded: pool 1–90 µg of protein, pool 2–155 µg of protein and pool 3–96 µg of protein) and the equivalent of 40 µg peptides was used for each TMT channel. Samples were digested using trypsin at 1:50 dilution at 37 °C overnight in 150 µl TEAB (final concentration 100 nM). Centrifugation at 4000 g for 30 s in 160 µl of 50 mM TEAB eluted peptides from S-trap mini spin column. Further centrifugations were performed in 160 µl of 2% aqueous formic acid and finally 160 µl of 50% acetonitrile/0.2% formic acid. Tryptic peptides from each sample eluate produced were pooled, dried and then quantified with Pierce Quantitative fluorometric Peptide Assay (Thermo Scientific). A TMTsixplex™ Isobaric Label Reagent Set (90,061) Pierce High pH Reversed-Phase Peptide Fractionation kit (Thermo Scientific, #84,868) was used to label samples with TMT following the manufacturer’s protocol. Desalted tryptic peptides for each sample were dissolved in 100 µl of 100 mM TEAB, then the 6 TMT labels were dissolved in anhydrous acetonitrile, added to the different samples and incubated for 1 h at room temperature. Labeling reaction was stopped with 8 µl of 5% hydroxylamine per sample. Following TMT labeling, samples were mixed, desalted and dried in speed-vac at 30 °C. Samples were then re-dissolved with 200 µl of ammonium formate (NH_4_HCO_2_) (10 mM, pH 9.5) and peptides were fractionated using High pH RP Chromatography. Two technical replicates were generated using the 3 pooled samples giving the 6-plex set up for TMT labelling. Full method as described [38]. Mass spectrometry analysis of the *post-mortem* samples was performed in the ‘FingerPrints’ Proteomics Facility at the School of Life Sciences, University of Dundee carried out as described [38]. In summary, peptide analysis was conducted using a Q-Exactive-HF (Thermo Scientific) mass spectrometer coupled with a Dionex Ultimate 3000 RSLC Nano (Thermo Scientific). Using the following buffers: buffer A (0.1% formic acid in Milli-Q water (v/v)) and buffer B (80% acetonitrile and 0.1% formic acid in Milli-Q water (v/v). Sample aliquots were loaded onto a trap column 10 μL/min (100 μm × 2 cm, PepMap nanoViper C18 column, 5 μm, 100 Å, Thermo Scientific) equilibrated in 0.1% formic acid and the column was washed for 5 min at the same flow rate. Column was then switched in-line with a Thermo Scientific, resolving C18 column (75 μm × 50 cm, PepMap RSLC C18 column, 2 μm, 100 Å). At a constant rate of 300 nl/min peptides were eluted from the column. A constant column temperature of 50 °C was maintained. Q-Exactive HF was run in data-dependent positive ionization mode. Voltage for the source was set to 2.4 kV with a capillary temperature of 250 °C. Mass accuracy was checked prior to before the start of sample analysis.

For the 6plex TMT analysis, the following Q-Exactive HF parameters were used. Full MS were acquired in a scan range of 335–1600 m*/z* at resolution 120 k and target values of 3 × 10^6^ charges with maximum IT set to 50 ms. In each scan cycle, the 15 most intense precursor ions were picked up at target values of 1 × 10^5^ charges and maximum IT set to 200 ms and fragmented using higher energy collision-induced dissociation (HCD) of 32. MS/MS spectra were recorded with a resolution of 60 k. Sequenced precursor masses were excluded from further selection for 45 s.

For experiments with hiPSC-derived MNs, protein extracts were precipitated with ice-cold acetone-methanol at -20 °C overnight. The proteins were pelleted by centrifugation (2000 × g, 20 min, 4 °C) and washed three times with 80% ice-cold acetone. Dried proteins were resolved in the digestion buffer (6 M urea, 2 M thiourea, 10 mM Tris, pH 8.0). For proteome analyses, 10 µg of proteins per sample were digested in solution with trypsin as described previously [93], and resulting peptides were desalted with C_18_ StageTips [60]. For the phosphoproteome, in each case one milligram of protein was digested as described above. Peptides were further purified on Sep-Pak 18 cartridges (Waters) and subjected to phosphopeptide enrichment by MagReSyn Ti-IMAC (ReSyn Bioscience) as described previously [33]. In brief, 100 μl of magnetic bead suspension per sample and enrichment round was washed with 70% ethanol, followed by washing with 1% NH_4_OH. Before peptide loading, beads were equilibrated with a loading buffer containing 1 M glycolic acid and 5% TFA in 80% ACN. Elution from the beads was performed three times with 1% NH_4_OH. The pooled eluates were further purified by C_18_ StageTips and peptides were subjected to two consecutive rounds of enrichment. LC–MS analyses of desalted peptides [60] were performed on an EASY-nLC 1200 UHPLC coupled to a Q Exactive HF, or quadrupole Orbitrap Exploris 480 mass spectrometer (all Thermo Scientific).

Separations of the peptides and enriched phosphopeptides were done as described previously [33, 93] with slight modifications: peptides were injected onto the column in HPLC solvent A (0.1% formic acid) at a flow rate of 500 nl/min and subsequently eluted with a 127 min (proteome) or 57 min (phosphoproteome) segmented gradient of 10-33-50-90% of HPLC solvent B (80% acetonitrile in 0.1% formic acid) at a flow rate of 200 nl/min. Both mass spectrometers were operated in a positive ion and data-dependent acquisition mode.

For the Q Exactive HF mass spectrometer, full MS were acquired in a scan range of 300–1650 m/z at resolution 60 k and target values of 3 × 10^6^ charges with maximum IT set to 25 ms. In each scan cycle, the 12 (proteome) or 7 (phosphoproteome) most intense precursor ions were picked up at target values of 10^5^ charges and maximum IT set to 45 ms and 220 ms, respectively, and fragmented using higher energy collision-induced dissociation (HCD) of 27. MS/MS spectra were recorded with resolution 30 k (proteome) and 60 k (phosphoproteome), respectively. In all proteome and phosphoproteome measurements, sequenced precursor masses were excluded from further selection for 30 s.

For the quadrupole Orbitrap Exploris 480 mass spectrometer, full MS were acquired in a scan range of 300–1750 m/z range at resolution 60 k with an automatic gain control (AGC) set to standard and a maximum ion injection time set to automatic. The 20 most intense precursor ions were sequentially fragmented with a normalized collision energy of 28 in each scan cycle using HCD fragmentation. MS/MS spectra were recorded with a resolution of 15,000, whereby fill time was set to automatic. In all measurements, sequenced precursor masses were excluded from further selection for 30 s.

### MS data processing

MS data were processed using default parameters of the MaxQuant software (v1.5.2.8, 1.6.2.10 or v1.6.14.0; a final analysis with the version 1.6.14.0 was performed to ensure reproducibility of the coverage across the analysis) [16]. Extracted peak lists were submitted to database search using the Andromeda search engine [17] to query a target-decoy [21] database of *Homo sapiens* (96,817 entries, downloaded on the 11th of December 2019) and 285 commonly observed contaminants.

In database search, full tryptic specificity was required and up to two missed cleavages were allowed. Carbamidomethylation of cysteine was set as fixed modification, whereas protein N-terminal acetylation, and oxidation of methionine were set as variable modifications. In addition, phosphorylation of serine, threonine, and tyrosine were set as variable modifications for phosphoproteome analysis. Initial precursor mass tolerance was set to 4.5 ppm, and 20 ppm at the fragment ion level. Peptide, protein and modification site identifications were filtered at a false discovery rate (FDR) of 0.01. The iBAQ (Intensity Based Absolute Quantification) and LFQ (Label-Free Quantification) algorithms were enabled, as was the “match between runs” option [44, 67].

The MS proteomics data have been deposited to the ProteomeXchange Consortium (http://proteomecentral.proteomexchange.org) via the PRIDE partner repository [55] and are available with the dataset identifiers PXD041543 (hiPSC-derivewd MNs) and PXD042617 (*post mortem* samples). Detailed information can also be found in Supplementary Table 2, online resource.

### Western blot

Western blots using human *post-mortem* samples were carried out as described [38]. Briefly, 10 µg of a sample with 4X Laemmli sample buffer (Bio-Rad, #1,610,747) and beta-mercaptoethanol was denatured for 5 min at 95 °C. Using 4–20% Tris–Glycine 1.0 mm polyacrylamide pre-cast gels (Termo Fisher, #WXP42020BOX), 5 µl protein ladder (Li-Cor #928–70,000) was loaded along with the 10 µg samples. Following electrophoresis, the transfer step was conducted using the iBlot™ 2 Gel Transfer Device (Invitrogen, #IB21001) and nitrocellulose membrane using precast transfer stacks (Invitrogen, #IB23001). Membrane was stained for total protein with REVERT™ 700 Total Protein stain (Li-Cor, REVERT™ 700 Total Protein Stain Kits, #926– 11,010) before being imaged with the Li-Cor Odyssey system. Membrane was destained post-imaging as instructed in the manufacturer's protocol and blocked with 5% milk/TBST or 5% BSA/TBST solutions for 1 h. After which, primary antibody solutions diluted in the appropriate block solution were added for 24 h. Following which the membrane was thoroughly washed, and the secondary antibody diluted in the block was applied for 1 h. After further washes, the membrane was imaged using the Li-Cor Odyssey system.

Western blots with samples from hiPSC-derived MNs were performed by resolving equal concentrations of protein (determined by Bradford Assay) on 10% acrylamide SDS-PAGE gels, which were then transferred to a nitrocellulose membrane using a Trans-Blot Turbo device (BioRad, USA). To block non-specific binding sites, the membranes were incubated with a 5% BSA solution (diluted in TBS pH 7.5 + 0.2% TWEEN) for two hours and incubated with the primary antibody overnight at 4 °C. Afterwards, blots were washed 3 times with TBS + 0.2% TWEEN, incubated with HRP-conjugated secondary Ab for 1 h, and again washed 3 times. Chemiluminescent signal was detected using the ECL detection kit (ThermoFisher Scientific, 32,106) and a MicroChemi 4.2 device (DNR Bio Imaging System). For quantification, Gel-analyzer Software 2010a was used. The values of the proteins of interest were normalized against the loading control, β-actin.

### Immunocytochemistry

Immunostainings were performed as previously described [14]. Following fixation with 4% paraformaldehyde (containing 10% sucrose), cells were first incubated with blocking solution (PBS + 10% Goat Serum + 0.2% Triton- × 100) for two hours at room temperature and subsequently with primary antibodies diluted in the same blocking solution overnight at 4 °C. After three washes with PBS (15 min each), the cells were incubated with secondary antibodies (diluted 1:1000 in PBS) for two hours at room temperature. Then, cells were washed again 3 times and mounted with ProLong™ Gold Antifade Mountant with DAPI (Invitrogen, P36935) and Ibidi Mounting Medium (Ibidi, 5001).

### Pharmacological treatment

The effect of Docosahexaenoic acid (Sigma-Aldrich D2534) treatment was tested on hiPSC-derived MN cultured in 24-well plates differentiated from the ALS cell lines. Treatment was carried out form DIV 21 until DIV 42, by replacing half of the medium every second day, using a final concentration of 100 µM. The effect of DHA treatment was determined by measuring the levels of the phenotypic rescue using immunofluorescence.

The Tetanus neurotoxin (Sigma-Aldrich T3194) was used at a final concentration of 15 nM. The treatment was performed in hiPSC-derived MN differentiated from the Healthy I cell line, starting from DIV 42 and carried out for 24 h.

### Primary rat cortical neurons

Primary cultures of rat cortical neurons were prepared from rat embryos (Sprague–Dawley rats, Janvier Laboratories) at embryonic day 18 as previously described [12]. Briefly, cerebral cortices were manually dissected under stereomicroscopic guidance. After 10 min of incubation with 0.25% trypsin–EDTA (Gibco), the tissues were washed three times with DMEM (Gibco) (containing 10% foetal bovine serum, 1% penicillin/streptomycin, and 1% GlutaMAX, henceforth referred to as DMEM +) and thus mechanically dissociated. Following a filtration through a 100 µm mesh filter, the dissociated cells were plated on poly-L-lysine-coated (Sigma-Aldrich) glass coverslips or plastic dishes and cultured in Neurobasal Medium (Gibco) (containing 1% P/S, 1% GlutaMAX and 2% B27 – henceforth NB^+^).

Poly-(GA)_175_-EGFP aggregates were over-expressed in primary cortical neurons using an AAV9 vector under the control of the human Synapsin 1 promoter to ensure selective neuronal expression. Viruses were produced by the Penn Vector Core (University of Pennsylvania, Philadelphia, USA). Transduction of neurons was performed at DIV 3 with either AAV9-hSyn-poly(GA)_175_-EGFP or AAV9-hSyn-EGFP (a gift from Bryan Roth; Addgene viral prep # 50,465-AAV9) as a control.

### Microscopy

Fluorescence microscopy was performed with a Thunder imaging system (Leica) equipped with a DFC9000 sCMOS camera, an HC PL Fluotar 20X air (N.A. 0.4) objective and using the LasX software (Leica).

Confocal microscopy was performed with a laser-scanning microscope (Leica DMi8) equipped with an ACS APO 63X oil DIC immersion objective. Images were acquired using the LasX software (Leica), with a resolution of 1024 × 1024 pixels and a number of Z-stacks (step size of 0.3 μm) encompassing the entire cell soma.

### Image analysis

To analyze the intensity levels of nuclear phospho-c-Jun^Ser63^ in immunostaining, the Z-stack was collapsed with the maximum intensity projection tool of ImageJ and the phospho-c-Jun^Ser63^ signal was measured within a region of interest (ROI) drawn using as a reference the neuronal nucleus (identified in the DAPI channel).

To analyze the intensity of SYP puncta, four ROIs (130 × 130 µm) were randomly drawn in each picture on the MAP2 channel to cover neuronal dendrites, then synaptic clusters were traced with the FindFoci plugin of ImageJ, using the Max Entropy algorithm.

The neuroprotective effect of the DHA treatment was assessed by assessing the number of neurons in the entire field of view acquired, using the MAP2 channel.

The same computational parameters and post-acquisition adjustments were used to analyze images from the same tests and to display figures.

### Antibody list

The following primary antibodies were used for Western Blot experiments with *post-mortem* samples: anti-Lamin (Proteintech, 10,298-1-AP; diluted 1:1000), anti-Synaptophysin (Abcam, ab8049; diluted 1:1000), anti-PSD95 (Cell Signaling, 3450; diluted 1:1000), anti-RTN3 (Proteintech, 12,055-2-AP; diluted 1:500), anti-TDP-43 (Abcam, ab133547; diluted 1:500) and anti-SerpinA3 (Proteintech, 12,192-1-AP; diluted 1:500). The secondary antibodies used were Donkey anti-Mouse IgG IRDye^®^ 800CW (Li-Cor, 923-32,212) or the Donkey anti-Rabbit IgG IRDye^®^ 800CW (Li-Cor, 926-32,213).

The primary antibodies used in experiments with hiPSC-derived MNs were: anti-MAP2 (Encor, CPCA-MAP2; diluted 1:1000), Proteostat^®^ aggresome detection kit (Enzo, ENZ-51035-0025; diluted 1:5000), anti-Synaptophysin (Abcam, ab14692; diluted 1:1000), anti-Synaptophysin (Synaptic Systems, 101 004; diluted 1:1000), anti-phospho-c-Jun (Ser63) (Cell Signaling, 91,952; diluted 1:1000), anti-phospho SQSTM1/p62 (Ser349) (Cell Signaling, 16,177; diluted 1:300). For Western blot experiments, the secondary HRP-conjugated anti-Mouse (1:3000 dilution) and anti-Rabbit (1:1000 dilution) antibodies from DAKO were used. For immunostainings, the following secondary antibodies from Thermo Fisher Scientific were used at 1:1000 dilution: goat anti-Rabbit Alexa Fluor^®^ 488 (A-11034), goat anti-Chicken Alexa Fluor^®^ 568 (A-11041), and goat anti-Guinea Pig Alexa Fluor^®^ 647 (A-21450).

### Lactate-dehydrogenase cytotoxicity assay

The CyQUANT™ LDH Cytotoxicity Assay (Thermo Fisher Scientific, Waltham, MA, United States; C20300) was used following manufacturer's instructions to measure the amount of cell stress and death within the cultures, by measuring the leaked LDH released from damaged cells in the culture medium. At DIV42, 50 µl of medium were taken from each culture and transferred to a 96-well plate kept at room temperature and protected from light. The medium was then mixed with 50 µl of the reaction mixture given with the kit. Additionally, 50 μl of Stop Solution (included in the kit) was added to each well after 30 min of incubation. Gen5 microplate reader (BioTek Instruments, Winooski, VT, United States) was used to measure the absorbance of the Formazan-dye produced by the reaction at 490 nm. To further detect and minimize background signals, the absorbance at 680 nm was measured and subtracted.

### qRT-PCR

Total RNA from hiPSC-derived MN was extracted using the RNeasy Mini kit (Qiagen) following the manufacturer’ instructions. First-strand synthesis and quantitative real-time-PCR amplification were performed in a one-step using the QuantiFast™ SYBR Green RT-PCR kit from Qiagen in a total volume of 20 µl. The primers used for qRT-PCR were purchased (Qiagen QuantiTect Primer Assays, Qiagen—validated primers without sequence information). The following settings were used: 10 min at 55 °C and 5 min at 95 °C, followed by 40 cycles of PCR for 5 s at 95 °C for denaturation and 10 s at 60 °C for annealing and elongation (one-step). The SYBR Green I reporter dye signal was measured against the internal passive reference dye (ROX) to normalize non-PCR-related fluctuations. The Rotor-Gene Q software (version 2.0.2) was used to calculate the cycle threshold values.

### Data and statistical analysis

Proteins identified in *post-mortem* mass spectrometry with less than two unique peptides were removed from the dataset (565 IDs). Similarly, proteins that were not identified in all samples were also excluded from the dataset (30 IDs). Following these data filtering steps, the dataset was analysis ready containing 5497 protein IDs. Reporter intensity value of each protein was log_2_ transformed and the group median was corrected to account for minor loading variables. An average of technical replicates per protein were counted for each group, then sALS and C9 + ve group were normalized to the control group providing the fold change value for each protein [1]. To visualize protein distribution by volcano plot, *p* value for each protein was generated using multiple t test with the original FDR method of Benjamini and Hochberg, then p values were -log_10_ transformed. Ratiometric values were generated by normalizing the average reporter intensity value of each protein of sALS and C9 + ve data to control than 1/group median corrected. Up – or down-regulated proteins were identified as a threshold of 20%-fold change (≥ 1.2 or ≤ 0.8 ratio change) [38]. This fold change threshold was chosen as post-hoc validation of protein expression with western blotting can be conducted with these levels of protein change.

Cell type-specific enrichment analysis of the post-mortem dataset was performed using different mouse and human cell type-specific gene clusters in the spinal cord [8, 87]. Cluster-specific proteins were selected and visualized by heat map using Z-scores.

To find synapse-specific TDP-43 interactors, a previously published TDP-43 whole-cell interactome dataset [69] was aligned with our post-mortem dataset. After the initial overlay, proteins with synaptic functions were selected using the Gene Ontology (GO) „Synaptic” term, revealing 854 protein IDs which were displayed by heat map.

Downstream statistical analysis of hiPSC-based proteomics and phosphoproteomics was performed in R (version 3.6.0). The R package proteus (version 0.2.14) was used to analyze MaxQuant’s Proteomics output file “proteinGroups.txt” and the phosphoproteomics output file “Phospho (STY)Sites.txt”. Differential expression (DE) analysis was performed with the R package Limma (version 3.42.2) outside of the package Proteus. As a cut-off for statistical significance, a nominal *p* value < 0.05 or adjusted *p* values (adj. *P*. Val.) < 0.05 were chosen. All data were quantile normalized to account for the variation of intensity between samples followed by log2 transformation.

*Phosphosites analysis in total MNs lysate*: To identify differentially expressed proteins/phosphosite a linear model was then fitted to each protein as follows: exp =  ~ condition with “exp” representing the expression of a protein and “condition” representing the factor of the experiment with the two levels of patient and control. The same procedure was done for phosphosites and afterwards the phosphosite table was compared against the protein table to exclude phosphosites that were different due to differential expression at the protein level. For graphical visualization we used a volcano plot to show statistical significance -log10(p-value versus log2 fold change).

*Proteomics analysis in hiPSC-derived MNs synaptosomal fraction*: To identify differentially expressed proteins a linear model was then fitted to each protein as follows: exp =  ~ X with “exp” representing the expression of a protein and “X” representing a combination of the main experimental factor health status with the levels patient and control, and specimen with the levels total lysate and synaptic fraction. For graphical visualization, we used a volcano plot to show statistical significance -log10(p-value) versus log2 FC.

Gene-centric analysis was performed by pooling the data obtained from mutant lines with mutations in the same gene and using as a significance cutoff 20%-fold change.

For the identification of KEGG pathways and Gene Ontologies (GO) that may be altered we used the g:Profiler (https://biit.cs.ut.ee/gprofiler/gost) or ShinyGo v0.75 online tools with the specification of Homo Sapiens datasets. The protein IDs corresponding to the DE phosphosites and proteins with nominal *p*-val < 0.05 were copied into the g:Profiler tool. Homo sapiens was selected as the species and for the advanced options the following parameters were considered: only annotated genes, g:SCS threshold, 0.05 threshold and ENTREZGENE_ACC; before clicking on Run query.

Enriched terms were downloaded and plotted using the ggplot2 package for R version 4.1.2 or GraphPad Prism (version 9.5.0). Venn diagram calculation and plotting were generated using the free online software of the Van de Peer Lab (https://bioinformatics.psb.ugent.be/webtools/Venn/). Heatmaps were plotted using hierarchical clustering with Euclidean distance by the heatmap.2 modules of the gpltots package of R or the Partek Flow softwares. Z-score was calculated as described before [38]. Protein–protein interaction networks were generated with the OmicsNet 2.0 software [91]. Kinome analysis was performed using the SELPHI tool [56] and the representation of the kinome tree was made using the KinMap tool (http://www.kinhub.org/kinmap/index.html).

To compare two independent groups (genotypes) in western blot, immunocytochemistry and LDH assay, we used an unpaired t test with Welch correction in the case of normally distributed data and a nonparametric Mann–Whitney test in the case of non-normal distribution. Here, experiments were performed in *n* = 3 replicates and normalized to the average value of the control group to obtain one single value for each cell line (representing a single patient). To analyze the effect of treatments performed in hiPSC-derived neuronal cultures, values obtained by analyzing cultures exposed to vehicle or treatment were compared using a paired t-test. Statistical significance was set at *p* < 0.05.

## Ethics approval

Patients were recruited through the Scottish Motor Neuron Disease Register, ethical approval was obtained from Scotland A Research Ethics Committee 10/MRE00/78 and 15/SS/0216. Use of patient samples for genetic profiling has been approved by the Chief Scientist Office Scotland; MREC/98/0/56 1989–2010, 10/MRE00/77 2011–2013, 13/ES/0126 2013–2015, 15/ES/0094 2015-present. *Post-mortem* human tissue was requested from the Edinburgh Brain Bank and approved by its ethics committee (21/ES/0087) and the ACCORD medical research ethics committee, AMREC (ACCORD is the Academic and Clinical Central Office for Research and Development, a joint office of the University of Edinburgh and NHS Lothian, approval number 15-HV-016).

All procedures with hiPSC material were in accordance with the ethical committee of the Ulm University (Nr.0148/2009 and 265/12) and in compliance with the guidelines of the Federal Government of Germany. All participants gave informed consent for the study. The use of human material was approved by the Declaration of Helsinki concerning Ethical Principles for Medical Research Involving Human Subjects.

The preparation of rat primary cells was allowed by the Permit Nr. O.103 of Land Baden-Württemberg (Germany), and performed in respect of the guidelines for the welfare of experimental animals issued by the German Federal Government and the Max Planck Society, and the ARRIVE guidelines.

## Results

### The spinal cord synaptic proteome is profoundly disrupted in ALS

**Fig. 1:**
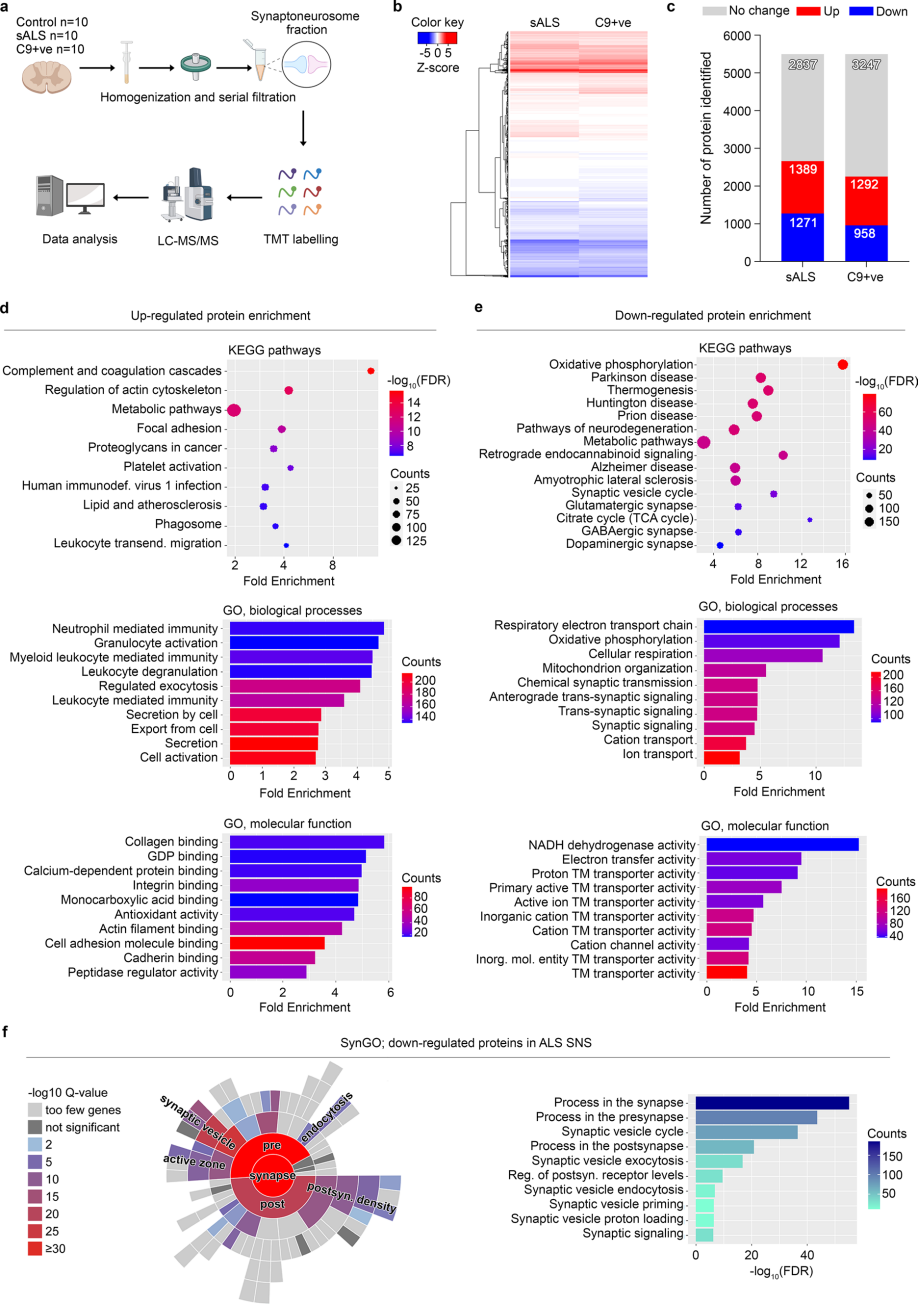
Enrichment analysis of the human ALS spinal cord synaptic proteome. **a** Schematic diagram shows the experimental workflow. Synaptically-enriched fractions were collected using fresh frozen human spinal cord tissue from age and gender-matched controls, sporadic ALS (sALS) and C9ORF72-RE + ve donors. After Tandem Mass Tag (TMT) labelling, proteome was examined using liquid chromatography, mass spectrometry-based quantification and identification (LC–MS/MS). Collected MS data were analysed using MaxQuant proteomics software. **b** Heatmap shows genotypic-specific protein changes using hierarchical clustering with Euclidean distance. **c** The number of up- and down-regulated proteins were counted using the control normalized ratiometric values on a threshold of 20% ≤ change in each direction. **d**, **e** Bioinformatics analysis revealed the top enriched KEGG pathways and Gene Ontology (GO) terms using the up- and down-regulated protein IDs. Colorized dot plots and bar graphs were created based on fold enrichment and gene counts/pathway. **f** Synaptic enrichment analysis using down-regulated protein IDs by the SynGO database shows enhancement in presynaptic terms including synaptic vesicle cycle

To investigate the synaptic proteome of the ALS spinal cord, we collected *post-mortem* samples from a total of 30 donors: 10 fALS patients carrying *C9orf72* repeat expansion (C9 + ve), 10 sporadic (sALS) with TDP-43 pathology and 10 age- and gender-matched healthy individuals. Samples from all donors were processed following published methodologies to extract synaptoneurosomes (SNS) [38]. Synaptic protein enrichment was assessed by western blot and confirmed significantly higher levels of the presynaptic protein Synaptophysin (SYP) and the postsynaptic scaffold protein, PSD95 in the SNS samples when compared to the total homogenate (TH, Supplementary Fig. 1a, online resource). These data, together with the absence of LaminA in the SNS, confirmed the efficient isolation of synapse-enriched fractions from the spinal cord samples. After sample quality control by total lysate staining to rule out major differences in tissue and protein degradation (Supplementary Fig. 1b, online resource), we followed a previously established pipeline [38] to analyze the total synaptic proteome using TMT-based mass spectrometry (Fig. 1a). We performed internal quality controls to ensure that the data obtained with mass spectrometry were truly representative of the original samples. First, we calculated the ratiometric change in the expression of PSD95, RTN, TDP-43, SERPINA3 and SYP based on western blot experiments. These were strategically selected as they represented proteins that either decreased, increased or did not change in ALS samples compared to controls, allowing us to confirm a dynamic range of protein alterations. Afterwards, we plotted these values against the proteomics ratiometric data and found a significant correlation (*R*^2^ = 0.9515, *p* = 0.0001; Supplementary Fig. 1c, online resource) between the two methods, suggesting the proteomics data accurately reflects changes within the original synaptic sample. Interestingly, the expression level of SERPINA3, TDP-43, RTN3, PSD95 and SYP were comparable in both ALS groups, suggesting similar disease-associated changes within this select panel of proteins, despite the different genetic background (Supplementary Fig. 1d, online resource).

In our MS experiments, we identified 5495 unique proteins present in all samples, 87% of which (4752 proteins) overlapped with a previous proteome obtained from total spinal cord lysate (Supplementary Fig. 2a, online resource) [54]. In addition, 85% of our dataset overlapped with 3 independent synaptic databases (SQLite, SynaptomeDB and SynGO; Supplementary Fig. 2b, online resource) [26, 36, 57, 75]. Together, this extensive bioinformatic analysis confirmed the strong synaptic enrichment in our proteomic data. By performing an enrichment analysis of the whole dataset using the ShinyGO online platform, we identified a significant overrepresentation of pathways linked to metabolism and neurodegenerative disorders (Supplementary Fig. 2c, online resource). In addition, GO analysis revealed that the identified proteins were mainly associated with mitochondria, synapse, vesicles, protein transport and localisation, as well as molecule binding (Supplementary Fig. 2d, online resource), further confirming the strong enrichment of synaptic material in our samples. Strikingly, we also identified the synaptic presence of 39 proteins coded by ALS pathogenic genes, whose levels were largely comparable in C9 + ve and sALS samples (Supplementary Fig. 2e, online resource). In agreement with previous studies, the C9ORF72 protein was found at the synapse and was most significantly reduced (by approximately 33%) in the C9 + ve group compared to the sporadic ALS group [38]. In addition, we found remarkable similarities in the altered proteome between the two ALS groups (Fig. 1b). In the sALS group we found 1389 up- and 1271 down-regulated proteins (> 20% compared to controls), while in the C9 + ve genotype there were 1292 up- and 958 down-regulated (Fig. 1c). Notably, the majority of the altered proteins showed the same direction of expression change in both the disease-associated groups, indicating a strong similarity in synaptic proteome alteration, regardless of genetic background. Because of this striking similarity and considering the selective MNs vulnerability in ALS, we first looked at the cellular composition within the spinal cord samples. We overlapped our dataset with publicly-available single-cell transcriptomics [8, 87] and identified cell-type specific markers revealing that the majority of the proteins specific for the different neuronal subtypes were indeed present in our *post mortem* specimens (Supplementary Fig. 3a–d, online resource). Importantly, we also could differentiate between alpha (vulnerable) and gamma (resistant) MNs [26, 36, 57, 75] (Supplementary Fig. 3e, online resource). This suggested that the selective loss of specific neuronal subtypes had a minor contribution to the synaptic alterations highlighted by our experiments and that the similarities between C9 + ve and sALS synaptome arise from molecular alterations rather than MNs death. The majority of the up-regulated proteins (1094) were indeed shared among both ALS groups (Supplementary Fig. 4c, online resource) and the top10 up-regulated molecules in each group, showed a similar trend in sALS and C9 + ve samples (Supplementary Fig. 4d, online resource). Enrichment analysis using only the smaller number of unique proteins up-regulated in each ALS group (shown in Supplementary Fig. 4c, online resource; sALS: 295, C9 + ve: 198) highlighted different pathways (Supplementary Fig. 4e–f, online resource). These group-specific changes may provide some clues about the unique aspects of these ALS sub-groups; however, the most striking feature is the collective overlap between protein changes across both experimental groups. A significant overlap between the two ALS-related synaptomes was indeed further identified in the set of differentially expressed and down-regulated proteins, which were 374 in the sALS and only 61 in the C9 + ve group (Supplementary Fig. 5a, online resource), with 897 proteins present as differentially expressed in both. Again, both ALS groups showed striking similarity in the top 10 down-regulated proteins (Supplementary Fig. 5b, online resource). While enrichment analysis with only the sALS-specific proteins (374 proteins) mainly highlighted metabolic alterations (Supplementary Fig. 5c, online resource), the low number of down-regulated proteins exclusively associated with C9orf72 mutations (61 proteins) did not lead to any significant pathway enrichment. Based on the strong similarity in synaptic alterations between sporadic and familial patients, we next focused on the comparison between both ALS groups and healthy controls.

TDP-43 pathology is a common hallmark of both sporadic and C9ORF72-RE patients and the sporadic patients were selected based on the presence of these aggregates. Given the fact that this protein is an important regulator of synaptic proteins and dynamics [84], we first searched for TDP-43 targets within our synaptic proteome. By aligning our dataset with a previously identified cluster of TDP-43 neuronal targets [69], we found that the majority of the proteins (4364) detected in our data could be potentially regulated by TDP-43. Within this large cluster, 864 molecules had a synaptic annotation and were significantly altered in the ALS groups (Supplementary Fig. 6a, online resource). In confirmation of the similarity between the C9 + ve and sALS synaptome, these 864 proteins displayed similar trends in both disease groups and the largest portion (including UNC13A) were down-regulated (Supplementary Fig. 6b and Supplementary Table 2, online resource). When we then looked at the total synaptic proteome, we observed a significant up-regulation of a broad range of pathways related to cellular structure, diverse molecular binding and inflammation (Fig. 1d), highlighting an increased inflammatory profile at the ALS synapse [78]. In contrast, the proteins down-regulated in the ALS synaptic fraction were predominantly associated with pathways linked to neurodegenerative diseases, mitochondrial function, synaptic signaling and diverse synapse subtypes (Fig. 1e), highlighting the presence of profound degenerative alterations within the spinal synaptic proteome of ALS patients. To better clarify which synaptic mechanism(s) might be mainly affected, we analyzed the down-regulated proteins using the online synaptic protein database, SynGO. In line with the loss of molecules required for “chemical synaptic transmission” and of mitochondrial proteins, which supply ATP at the synapse [73], SynGO analysis revealed a strong representation of mechanisms related to the release of synaptic vesicles (Fig. 1f). This highlighted a significant disruption in presynaptic vesicle dynamics in ALS.

### Analysis of the synaptic proteome from hiPSC-derived MNs highlights presynaptic alterations in ALS

**Fig. 2:**
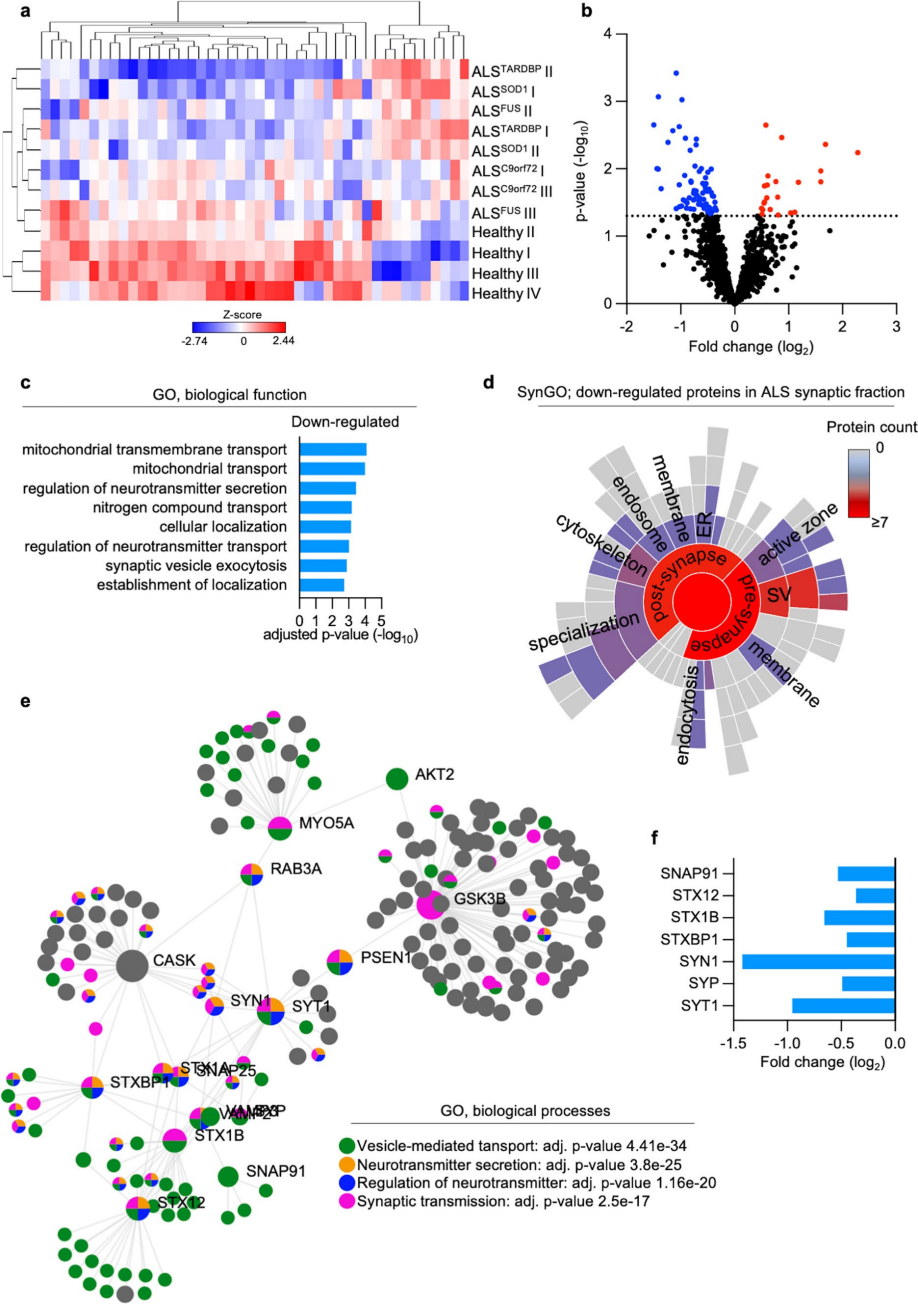
Synaptic proteome from hiPSC-derived MNs highlights presynaptic alterations in ALS**. a** Heatmap showing the significantly altered proteins identified in the ALS synaptic fraction compared to the control group. **b** Volcano plot represents the differential levels of the significant synaptic proteins in ALS MNs, with most of them being down-regulated. **c** ALS-specific enrichment based on down-regulated synaptic proteins reveals GO (biological function) terms linked to mitochondria and presynaptic function. **d** SynGO analysis reveals the sub-synaptic fractions (cellular component) significantly altered in ALS. **e** Protein–protein analysis based on the down-regulated molecules n ALS identifies a network of proteins significantly related to synaptic vesicles and neurotransmitter release. **f** Crucial members of the SNARE machinery are down-regulated in ALS

We then aimed to investigate the mechanisms behind the synaptic alterations discovered in our ALS *post-mortem* work, using an independent human model. Based on our previous work [74], we differentiated human iPSCs (hiPSCs) from fALS patients and healthy controls into spinal MNs. After 6 weeks in vitro (DIV42), we isolated the synaptic subcompartments from MNs harboring mutations in the *C9orf72, FUS, SOD1* and *TARDBP* genes (2 patients each) as well as 4 controls (Table 2) following a standardized protocol [59]. We then pooled the pre- and postsynaptic fractions (Supplementary Fig. 7a, online resource) to obtain a sample containing both sides of the neuronal contacts before performing mass spectrometry. First, by comparing the synaptic proteome to the total homogenate (Supplementary Fig. 7b, online resource) of all the samples independently from their genotype by using SynGO, we confirmed that the synaptic fractions were indeed significantly enriched in protein belonging to this neuronal microenvironment (Supplementary Fig. 7c, online resource). We then looked at the synaptic changes comparing ALS to the control group (Fig. 2a) and noticed that most of the significantly altered proteins were down-regulated in the ALS cultures (Fig. 2b). Interestingly, and in agreement with the data obtained from *post-mortem* material, the significantly reduced proteins in ALS clustered into GO terms linked to mitochondrial dynamics and neurotransmitter release (Fig. 2c and Supplementary Fig. 8, online resource). Of note, the up-regulated molecules did not significantly cluster in any pathway annotated within the biological processes category. We then aimed to better understand the synaptic function of the molecules that were significantly down-regulated in ALS MNs. While the proteins with previous SynGO annotations at the postsynapse were mainly associated with structure and organelles (endoplasmic reticulum and endosomes), the synaptic ontology tool confirmed a strong impairment in the machinery associated to synaptic vesicle and release of neurotransmitter (Fig. 2d). Indeed, enrichment analysis highlighted a network of proteins strongly connected with the down-regulated ones in ALS (Fig. 2e), which represented some of the key members of the SNARE machinery such as SNAP91, STX12, STX1B, STCBP1, SYT1, SYP and SYN1 [61]. To confirm this profound alteration affecting the SNARE complex, we investigated the pure S3 (presynaptic) fraction obtained from independent cultures of ALS and control MNs. Western blot analysis revealed a significant reduction of the presynaptic levels of SYP in all the mutant lines when compared to the four controls, thus confirming that impaired vesicle release is a major phenotype of ALS-related synapses (Supplementary Fig. 9, online resource). Importantly, SYP levels were also significantly reduced in our human ALS *post-mortem* synapses (Supplementary Fig. 1). Interestingly, despite the shared presynaptic pathomechanism across all of the ALS MNs, PCA analysis clearly separated the ALS MN subtypes into two clusters based on their mutation-specific characteristics (Supplementary Fig. 10a, online resource). To unfold the mutation-specific protein changes more precisely, we aligned the up- and down-regulated proteins from each cell line. There were small numbers of up-regulated proteins, and they showed segregation between the different ALS MN subtypes. The small number of up-regulated proteins in the *TARDBP* mutants did not provide any enrichment (Supplementary Fig. 10b, online resource). *C9orf72*-specific up-regulated protein change revealed the involvement of the TOR pathway (Supplementary Fig. 10c, online resource), meanwhile *FUS* and *SOD1* shared common features in terms of RNA processing and neurodegenerative diseases-related pathways (Supplementary Fig. 10d-e, online resource). Not surprisingly, as indicated above, the majority of the down-regulated proteins were shared across the ALS MN subtypes (Supplementary Fig. 11a, online resource). Interestingly, *SOD1* mutation-specific changes in down-regulated proteins mostly grouped in neurodegenerative-related pathways but did not reveal any specific enrichment in GO terms (Supplementary Fig. 11b, online resource). Although down-regulated proteins specifically identified in *FUS* and *C9orf72* mutants shared enrichment in the lysosomal pathway, *C9orf72* also shared some common terms with *TARDBP* mutant lines regarding alteration in cellular respiration and different transportation processes. In addition, FUS-specific down-regulated proteins were enriched in terms of immune responses and exocytosis (Supplementary Fig. 11c-e, online resource). However, the most striking finding from these analyses was represented by the strong congruency in the downregulation of presynaptic vesicular machinery across all mutant ALS MNs and human post-mortem spinal cord samples.

### Integration of *post-mortem* and hiPSC-derived MNs synaptosomal data identifies pathologic alterations in the vesicle release machinery

**Fig. 3:**
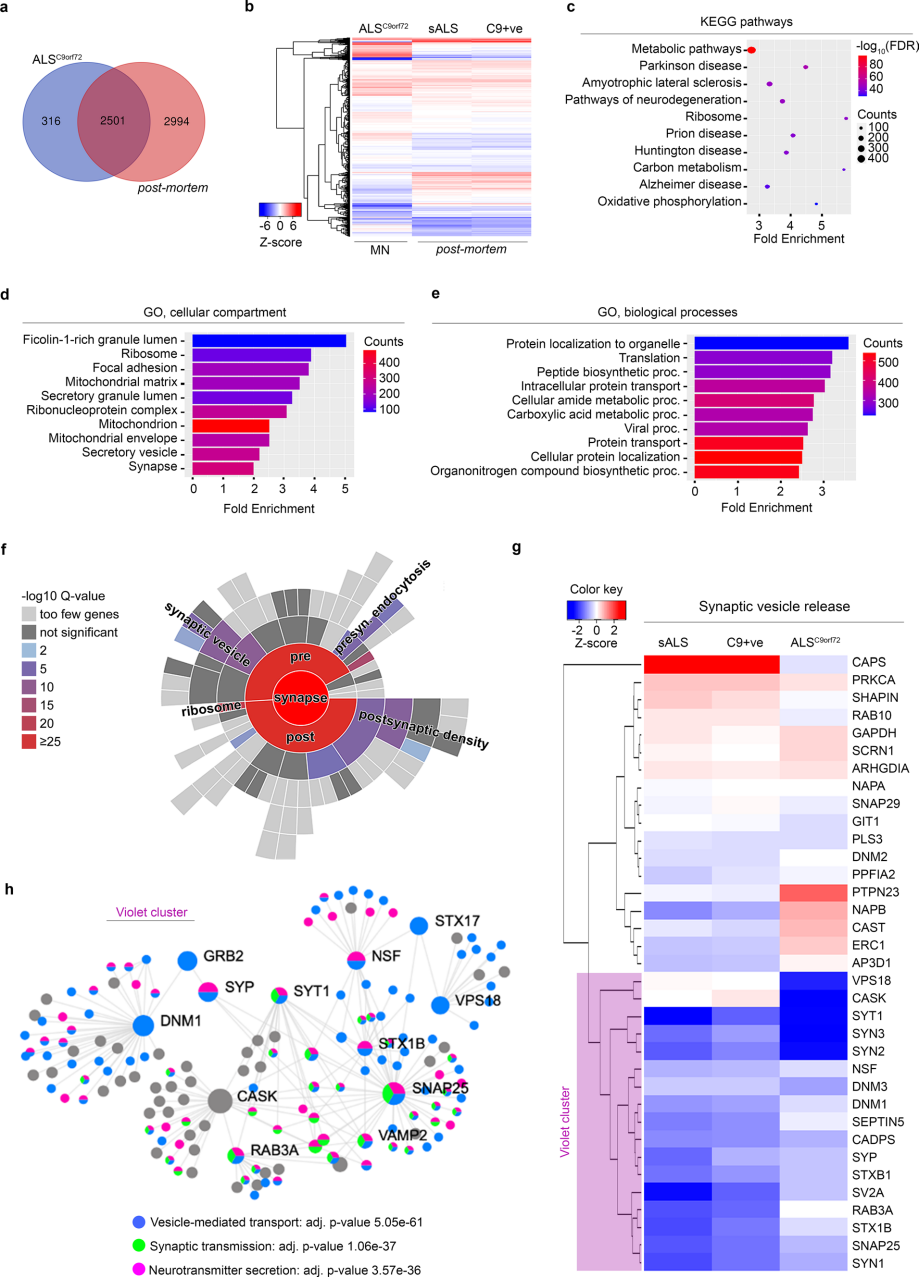
Alignment of the human *post-mortem* and hiPSC c9orf72 cell line proteomics dataset reveals synaptic vesicular dysfunction. **a** C9orf72 hiSC cell line ALS^C9orf72^ shows 89% overlap with the human *post-mortem* dataset. **b** Heatmap demonstrates ALS group-specific protein changes using the 2501 congruent proteins. **c-e** Aligned dataset were used for further enrichment investigation. Based on fold enrichment and gene counts (dot size) top 10 KEGG pathways (**c**) were visualized by colorized dot plots. Different Gene Ontology (GO) terms (**d-e**) plotted by fold enrichment and the bar graph is colorized by gene number/pathway. **f** Synaptic enrichment analysis was created using SynGO database, revealing enriched synaptic functional terms such as synaptic vesicle cycle, presynaptic endocytosis and postsynaptic density. **g** Heatmap shows specific protein changes in terms of synaptic vesicle release. **h** Protein–protein interaction network built on the violet cluster identified in **g**

Analysed independently, it was apparent that our *post-mortem* and hiPSC synaptic data were revealing similar changes in presynaptic machinery. We, therefore, aimed to more accurately identify convergent synaptic alterations between the *post-mortem* and the hiPSC proteomes. First, we assessed the similarities across datasets without considering the different pathogenic mutations. The majority of the molecules identified in the in vitro model were also present in the *post-mortem* samples, even though we could detect a larger number of synaptic proteins in the latter ones (Supplementary Fig. 12a, online resource). HiPSC-derived MNs have a synaptic transcriptome closer to the embryonal one [74] and, accordingly, our analysis of the significantly altered proteins in the ALS groups highlighted a separation between in vitro and *post-mortem* samples (Supplementary Fig. 12b, online resource). Still, enrichment analysis based on the commonly expressed proteins showed significant enrichment in KEGG terms associated with neurodegeneration, metabolism, proteasome and ribosome (Supplementary Fig. 12c, online resource), which confirmed the presence of underlying common pathological alterations within the ALS synaptic proteomes. Bioinformatics analysis revealed a heterogeneous group of enriched pathways when overlapping *in-vitro* and *post-mortem* proteins, including catabolic, metabolic and binding processes as well as different sub-synaptic compartments (Supplementary Fig. 12d-f, online resource). Nevertheless, SynGO analysis identified significant synaptic alterations associated with postsynaptic density, endocytosis and, most notably, nearly 50 proteins associated with the synaptic vesicles cycle (Supplementary Fig. 12 g, online resource). Despite the heterogeneity of this combined dataset, our data does reveal a consistent alteration of the presynapse, suggesting that dysfunctional synaptic vesicle machinery might play a crucial role in ALS. To gain more precise information on this aspect of the pathology and reduce some of the noise introduced by a diverse set of genetic backgrounds, we focused on the ALS^C9orf72^ genotype by comparing this specific sub-group of the ALS MNs to the *post-mortem* samples. Again, the majority of the synaptic proteins identified in vitro were also detected in the *post-mortem* samples (Fig. 3a) and the hierarchical clustering separated the cultured cells from patients´ biopsies (Fig. 3b). Enrichment analysis highlighted a significant representation of KEGG terms associated with neurodegeneration and others similar to those found when comparing the two larger, collective datasets (Fig. 3c). This indicated that the synaptic proteome of *C9orf72*-mutant MNs might largely represent the alterations observed in heterogeneous cases of ALS at this neuronal subcompartment. In particular, GO analysis highlighted a significant enrichment in terms linked to mitochondria, synapse and metabolic processes (Fig. 3d–e), while SynGO identified synaptic vesicles release as a significantly represented term (Fig. 3f). When we looked at the protein clustered in this pathway in detail (Fig. 3g), we noticed that a cluster of SNARE proteins (violet cluster) were down-regulated in all the samples. Protein–protein interaction analysis with the members of the violet cluster, which included SYP, SYN1, SYN2, SYN3, SYT1 SNAP25 and other key SNAREs, revealed a strongly significant enrichment in vesicle and neurotransmitter secretion (Fig. 3h). Thus, our data indicate that altered release of presynaptic vesicles represents a crucial synaptic phenotype in the ALS spinal cord, which is reliably recapitulated in cultured hiPSC-derived ALS MNs.

### Analysis of the ALS phosphoproteome links synaptic disruption to aggregates accumulation and Jun misactivation

**Fig. 4:**
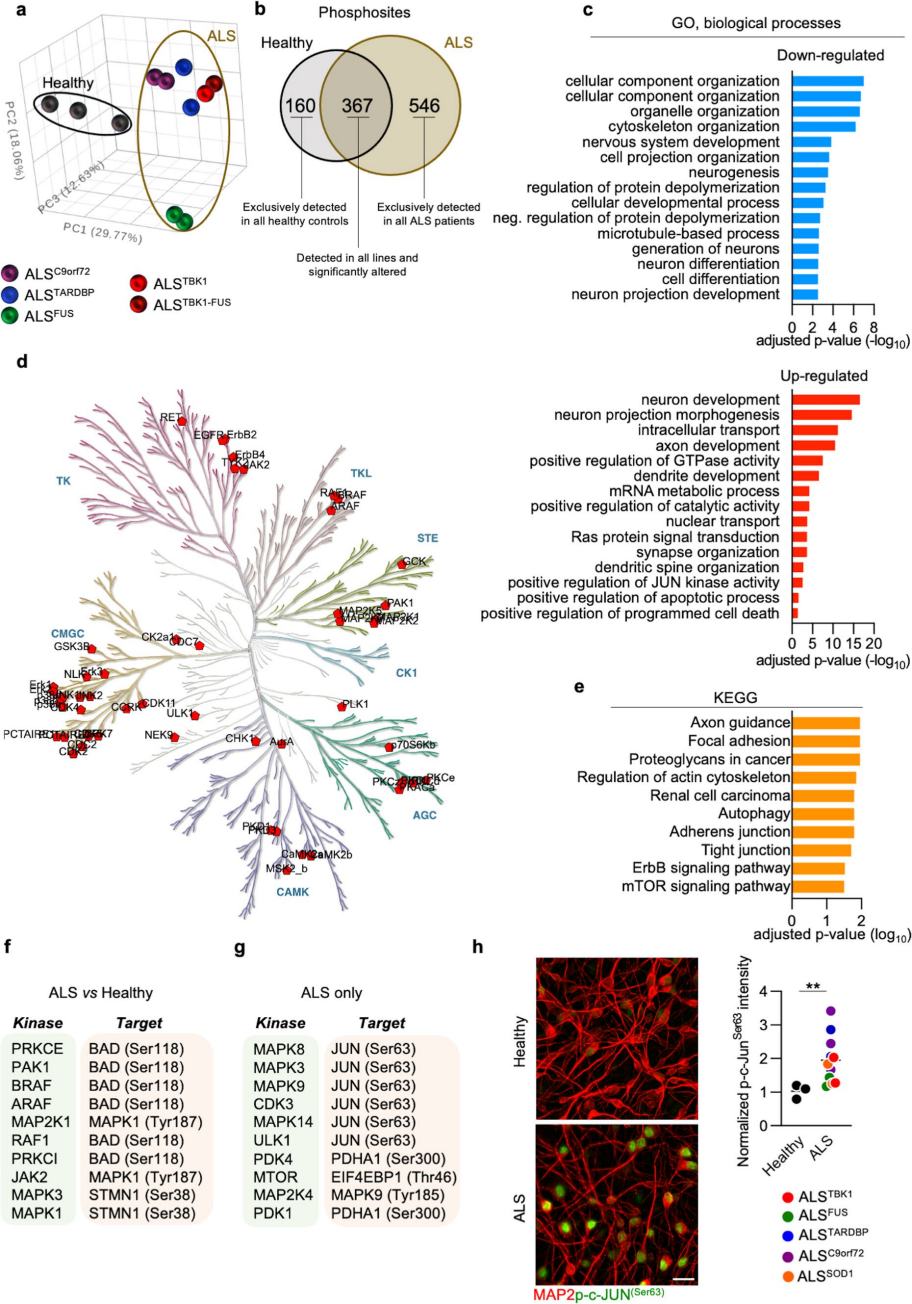
Phosphoproteome analysis highlights convergent JUN activation in ALS.** a** Principal Component Analysis (PCA) of the normalized expression values showing separation between the healthy and ALS phosphoproteomes. **b** Venn diagram shows the unique and shared phosphosites in both genotypes. **c** Enrichment analysis displaying the of the top down- and up-regulated GO (biological processes) terms in ALS. **d**, **e** Kinome tree and top enriched KEGG pathways using predicted kinases linked to the ALS phosphoproteome. **f**, **g** Top 10 significantly enriched kinases (and their targets) in ALS MNs. **h** Immunocytochemistry confirms that ALS MNs have significantly higher nuclear levels of phospho-c-Jun^Ser63^ than healthy controls at DIV 42. Welch's *t*-test **P < 0.01. *n* = 3 independent cultures. Scale bar: 25 µm

Besides allowing the propagation of electric signals, synaptic transmission triggers the downstream activation of a wide range of intracellular signaling cascades based on subsequent target phosphorylation reactions. Thus, we reasoned that the phosphoproteome of mutant MNs could reveal the mechanistic link between synaptic alterations and their vulnerability in ALS. To this end, we differentiated human MNs and cultured them for 6 weeks before performing mass spectrometry to analyze the phosphoproteome of ALS and healthy cultures. In this approach, we included the same ALS^C9orf72^, ALS^TARDBP^ and ALS^FUS^ lines as in the analysis of the synaptic proteome, and incorporated two *TBK1*-mutant hiPSCs lines (one carrying a compound *FUS* mutation and referred to as ALS^TBK1−FUS^). Since *TBK1* represents an important pathogenic gene whose protein product phosphorylates crucial targets in ALS-related pathways [13], by including these mutant MNs we reasoned to strengthen the outcome of the MS investigation. We readily observed a net separation of the ALS phosphoproteome from the healthy one (Fig. 4a and Supplementary Fig. 13a, online resource), confirming the presence of underlying alterations commonly characterizing the ALS phosphoproteome. Out of the 12,071 non-redundant phosphosites identified (Supplementary Fig. 13b, online resource), the majority were up-regulated in the mutant genotype (Supplementary Fig. 13c, online resource). We then considered for further analysis the 367 phosphosites identified in all the lines and having an adjusted *p*-value ≤ 0.05 (out of the 6142 proteins identified in all lines), as well as the 160 found only in all the control lines (considered as down-regulated in ALS) and the 546 exclusively found in all the eight mutants (considered as up-regulated in disease) (Fig. 4b). Enrichment analysis revealed that the down-regulated phosphorylation sites in ALS occurred on proteins mainly involved in GO *biological processes* linked to cellular structure and development, as well as neuronal differentiation and projection (Fig. 4c), while GO *cellular component* terms linked to the synapse were significantly down-regulated when compared to Healthy controls. Interestingly, we also found that the proteins with higher phosphorylation levels in ALS than in Healthy MNs clustered in terms associated with neuronal structures like the axon, dendrite and synapse (Fig. 4c and Supplementary Fig. 14, online resource). This highlighted that neuronal structures, such as synapses, are affected in ALS also at the phosphoproteome level. Accordingly, SynGO analysis of the synaptic alterations linked to the phosphoproteome of ALS cultures highlighted a significant enrichment in pathways linked to synapse organization, signaling and transmission (Supplementary Fig. 15, online resource), revealing that the ALS synapse is affected not only in the machinery of vesicle release but also in its phosphoproteome. In addition, we identified increased catabolic processes, Jun-related signaling and apoptosis in the ALS genotype, suggesting that synaptic and phosphoproteomic aberrations jointly contribute to MNs degeneration (Fig. 4c).

Interestingly, the aberrant phosphoproteome detected in the ALS genotype appeared to be mostly shared across the different pathogenic genes (Supplementary Fig. 16a, online resource). In fact, gene-centric analysis revealed a minor number of phosphosites specifically up-regulated in the different ALS sub-groups, which mainly enriched in terms linked to cytoskeleton and cellular component organization in ALS^C9orf72^ MNs (Supplementary Fig. 16b, online resource), while ALS^FUS^ cultures showed increased metabolic processes and adherens junction (Supplementary Fig. 16c, online resource). Of note, the proteins selectively up-regulated in ALS^TARDBP^ and ALS^TBK1^ did not significantly cluster in any of the ontologies investigated. In line with these findings, we identified minor gene-specific alterations also when analysing the down-regulated phosphosites in ALS when compared to controls (Supplementary Fig. 17a, online resource). While ALS^C9orf72^ MNs selectively showed reduced pathways involved in actin and cytoskeleton organization (Supplementary Fig. 17b, online resource), mutations in *FUS* and *TARDBP* led to reduced levels of different phosphosites that still converged toward cellular localization and mRNA surveillance mechanisms (Supplementary Fig. 17c-d, online resource). In contrast, the uniquely down-regulated proteins in ALS^TBK1^ cultures were mainly involved in cellular and biological processes, as well as GTPase activity (Supplementary Fig. 17e, online resource). All in all, these data highlight that despite the heterogeneity of the underlying mutations, which still lead to gene-specific alterations, ALS mutants strongly converge toward a unifying phosphoproteomic signature.

We then analyzed the kinome linked to the ALS phosphoproteome using the SELPHI tool [56], which highlighted that most of the predicted kinases clustered in the proline-directed serine/threonine kinase (CMGC, brown arm of the tree), tyrosine kinase (TK, purple arm of the tree) and serine/threonine kinase (STE, green arm of the tree) families (Fig. 4d). We also found a significant enrichment in kinases involved, among others, in axon guidance, autophagy and mTOR pathway, as well as ErbB signaling (Fig. 4e). When we compared the datasets associated with the ALS and Healthy genotypes, the phosphorylation of BAD at serine 118 was the top predicted target in mutant MNs (Fig. 4f). In addition, analysis based on the phosphosites identified only in the disease group predicted phospho-c-Jun^Ser63^ as the most phosphorylated site, mainly as a target of mitogen-activated protein kinase (MAPK) family members (Fig. 4g). We then investigated the levels of the kinases predicted with SELPHI in our *post-mortem* samples and found that 6 of them were up- and 8 down-regulated in both sALS and C9 + ve samples (Supplementary Fig. 18, online resource). In particular, MAPK14 showed the highest up-regulation, suggesting a mechanistic link between synaptic alterations, c-Jun phosphorylation and neuronal stress. These proteins are indeed associated with apoptosis and, accordingly, ALS MN cultures showed higher levels of leaked LDH than control ones (Supplementary Fig. 19, online resource). The increased neuronal sufferance was confirmed by the misactivation of c-Jun signaling observed in all the mutant lines of our library by immunocytochemistry (Fig. 4h), indicating that increased phosphorylation of c-Jun represents a common manifestation of the pathobiochemistry associated with ALS. In fact, this phenotype was prevented by correcting, with CRISPR-Cas9, the pathogenic GGGGCC expansion in one of our *C9orf72*-mutant lines (Supplementary Fig. 20, online resource).

We then sought to identify a possible mechanistic link between the presynaptic alterations and the increased activation of pro-apoptotic signaling mediated by Jun that we detected in ALS. We previously showed that accumulation of cytotoxic aggregates alters CREB-dependent transcription of synaptic genes in *C9orf72*-mutant MNs [14] and our phosphoproteomic data highlighted increased levels of phospho-SQSTM1/p62^Ser349^ in ALS cultures (Supplementary Table 3, online resource). Since SQSTM1/p62 is an aggregation-prone protein that gets massively sequestrated within toxic aggresomes [14], we speculated that the accumulation of these aberrant structures might represent a common pathologic feature triggering the biochemical abnormalities here identified and shared across the ALS spectrum. In line with the phosphoproteomic data, we found that all the mutant cultures were characterized by the accumulation of cytosolic toxic aggresomes in which phospho-SQSTM1/p62^Ser349^ was trapped (Supplementary Fig. 21, online resource). We then aimed at clarifying whether the accumulation of such aberrant structures might be sufficient to induce the same alterations observed in our models. To this end, we overexpressed the *C9orf72*-related dipeptide repeat (DPR) protein poly(GA) using an AAV9 vector under the control of the human synapsin promoter [14] in wild-type rat primary cortical neurons. Overexpression of poly(GA), which is known to sequester SQSTM1/p62 as well [47], significantly increased the levels of phospho-c-Jun^Ser63^ (Supplementary Fig. 22a, online resource) and reduced the levels of SYP (Supplementary Fig. 22b, online resource) in comparison to EGFP-expressing neurons.

Since the accumulation of aggregates appeared to be sufficient for inducing presynaptic impairment and activating stress signals converging on Jun, we aimed at better dissecting the interplay between these events contributing to MNs degeneration. We found that inhibition of synaptic vesicle release with tetanus toxin (TeNT) could enhance the levels of phospho-c-Jun^Ser63^ in Healthy MNs at values significantly higher than vehicle-treated ones (Supplementary Fig. 23, online resource). Thus, impaired presynaptic activity is sufficient to trigger Jun misactivation.

### Neuroprotection by docosahexaenoic acid rescues synaptophysin levels, reduces Jun activation and increases MN survival across the fALS spectrum

**Fig. 5:**
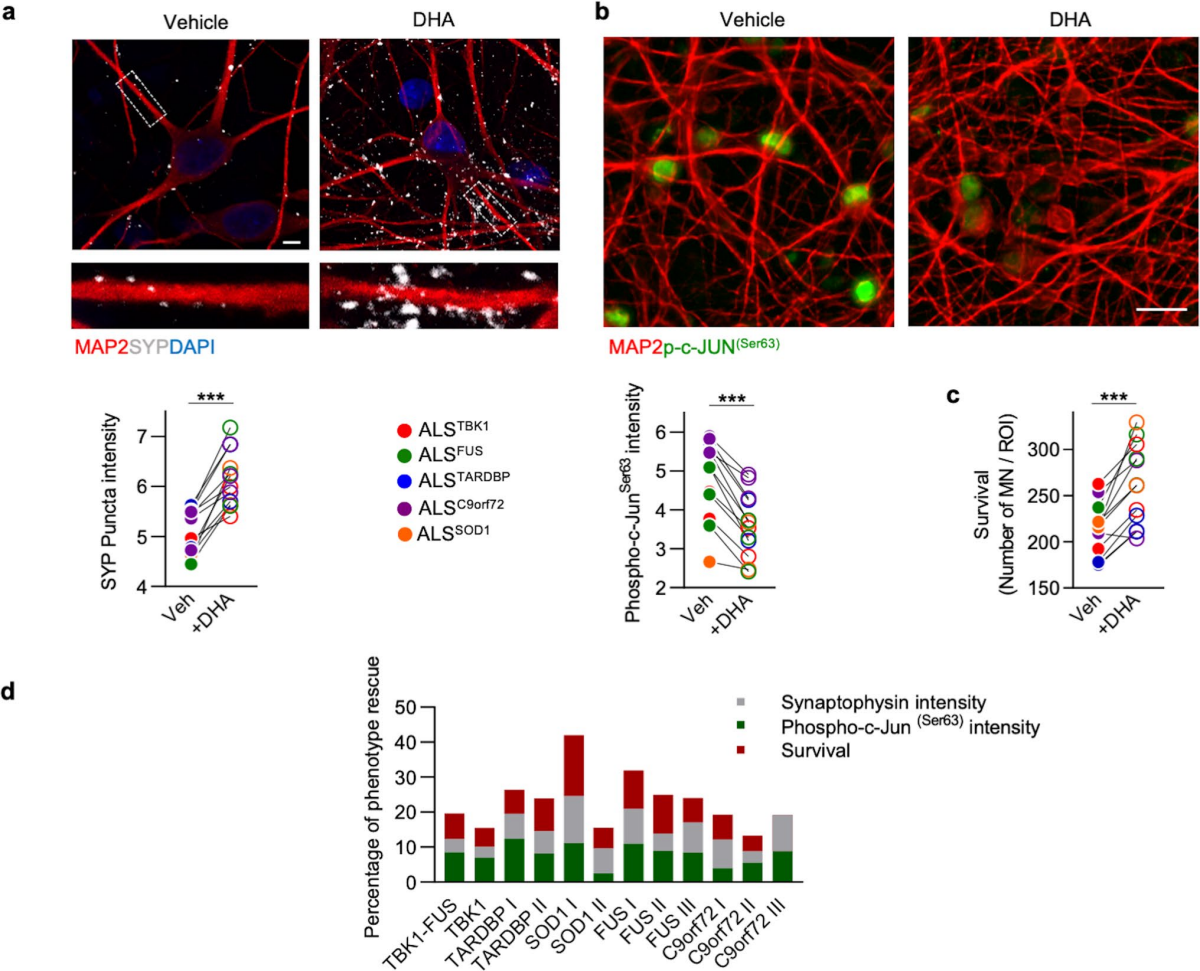
Docosahexaenoic acid's neuroprotective effects in fALS. **a** Representative images and quantitative data for the impact of DHA on the SYP levels in 12 fALS cell lines. DHA treatment significantly increases SYP intensity in all treated cell lines. Paired t-test ***P < 0.001. *n* = 3 independent treatments with each hiPSC line. Scale bar: 5 µm. **b**, **c** Treatment with DHA exerts a neuroprotective effect in fALS cultures by decreasing the levels of p-c-Jun^Ser63^ and improving MN survival in mutant cultures. Paired t-test ***P < 0.001. *n* = 3 independent treatments with each hiPSC line. Scale bar: 25 µm. **d** Evaluation of DHA efficacy in 12 fALS cell lines with multiple ALS-related phenotypes. *n* = 3 independent treatments with each hiPSC line. To adjust the maximum value of phenotype rescue to 100% when accumulating three endpoints, the upper limit of phenotype rescue for each item was compressed to 33.3%

Next, we hypothesized that the re-establishment of the presynaptic molecular composition might provide *proof-of-principle* evidence for the identification of neuroprotective targets across the ALS spectrum. Since the catabolic alterations leading to the pathophysiological accumulation of aggregates differ according to the underlying mutated genes [82], we speculated that interventions at the presynaptic levels (i.e., SYP) might circumvent the heterogeneity of the ALS lines analyzed. Since the presynapse represents the terminal part of the axon, whose integrity and dynamics are dramatically impaired across the ALS spectrum (Fig. 4e) [39], we speculated that improving axonal integrity might represent a valid strategy to contrast the presynaptic phenotype identified in this study. Docosahexaenoic acid (DHA) is a member of the omega-3 fatty acid family and represents a crucial component of the cellular membrane within the CNS [29]. It has been shown that docosahexaenoic acid (DHA) can modulate axonal dynamics [51] and positively impact recovery after spinal cord injury, with its neuroprotective effects including a significant increase in SYP levels [43]. Notably, DHA levels have also been found to be reduced in the spinal cord of ALS patients [30], and when dietary intake of DHA is reduced in rodents, several key presynaptic proteins involved in vesicle dynamics are reduced [72]. On the basis of these evidences, we set out to investigate the potential neuroprotective effect of this polyunsaturated fatty acid (PUFA) in our MNs cultures. First, we evaluated the effect of sub-chronic DHA treatment on control MNs. At a concentration of 100 µM, DHA did not alter the activation of c-Jun in control cultures (Supplementary Fig. 24a, online resource), indicating the absence of toxic effects. In contrast, and in support of our treatment choice, we detected a significant increase in the levels of dendritic SYP in cultures treated with fatty acid in comparison to vehicle-treated ones (Supplementary Fig. 24b, online resource). Based on these results, we exposed mutant MNs, differentiated from all the patients´ hiPSC-lines in our library, to 100 µM DHA for 3 weeks. As observed in healthy neurons, the intensity levels of SYP were significantly higher in the treated cultures than in untreated ones (Fig. 5a), thus confirming the effect of the fatty acid on this presynaptic protein. Notably, DHA-mediated elevation of SYP correlated with significantly lower activation of c-Jun (Fig. 5b), suggesting a neuroprotective effect exerted by the treatment in all the mutant lines. In fact, treating ALS-related MNs with DHA significantly reduced the leakage of LDH (Supplementary Fig. 25a, online resource), as well as the expression of the pro-apoptotic genes *AEN, CASP3* and *CFLAR* (Supplementary Fig. 25b–d, online resource), whose expression was predicted to be controlled by Jun using the *Harmonizome* software [62]. Accordingly, DHA-treated cultures displayed a significantly higher MN survival than those exposed to vehicle (Fig. 5c).

Thus, even though with different magnitudes (Fig. 5d), DHA rescued the synaptic levels of SYP and the pro-apoptotic activation of the transcription factor c-Jun in a heterogeneous cohort of fALS-related MNs, providing crucial information on how convergent pathomechanisms might be beneficially targeted across the ALS spectrum.

## Discussion

Alterations at the neuromuscular junction (NMJ) have historically been the most investigated synaptic dysfunction in motoneuron disease. Since patients experience a progressive loss of muscle innervation before eventually dying from respiratory failure, the NMJ has been identified as a crucial structure involved in the neurodegenerative dynamics characterizing this disease. Nevertheless, motor symptoms become obvious only after denervation and, in fact, muscular weakness and atrophy are used as prognostic indicators in the symptomatic phase of the pathology. While this represents an advantage for the clinical evaluation of its progression, it still leaves unanswered whether synaptic alterations might occur before the advanced stages of neurodegeneration. Interestingly, recent evidence from the SOD1^G93A^ mouse model highlighted that the vulnerable fast-fatigable MNs display reduced excitability and altered firing properties before muscle denervation [45]. This suggests that alterations within the excitatory neuronal network of the spinal cord might anticipate NMJ degeneration and symptoms. In agreement with this, synaptic abnormalities have been shown also in local interneurons at the early stages of the disease before the detection of MN death. This contributes to a dramatic inhibition/excitation imbalance that has been described as causative of MN hyperexcitability and NMJ disruption [3, 15]. Since hyperexcitability appears to be a feature mainly characterizing disease-resistant MNs [40], it is reasonable to speculate that the loss of excitatory inputs to vulnerable MNs might represent the trigger of a pathological chain of events leading to the loss of this specific neuronal population. Accordingly, dysfunctional presynaptic terminals belonging to the Ia afferents have been observed at the early stages of ALS and spinal muscular atrophy (SMA) [4, 50], providing evidence for a central role played by excitatory synaptic inputs in defining MN vulnerability.

Together with a local synaptic imbalance, recent observations have postulated a corticofugal theory of disease spreading from the neocortical areas to the spinal cord [9]. Here, the anterograde trans-synaptic spread of phosphorylated-TDP43 [22], as well as of C9orf72-related dipeptide repeats [83] and the synaptic accumulation of FUS [17], has been suggested to correlate with the progressive worsening of the motor symptoms observed in patients. Thus, it seems that the sensory afferents and corticospinal inputs might both play key roles in disease progression and MN loss, indicating that spinal synapses are crucial in disease progression. To better elucidate this aspect of neuronal vulnerability within the ALS spinal cord, we applied integrative proteomics combined with patient-derived models. These novel datasets mapping the spinal synaptic proteome in humans highlighted dramatic synaptic alterations in ALS. Interestingly, the magnitude of synaptic aberrations occurring within the spinal cord appears to be significantly larger than in cortical areas of ALS brains with comparable approaches [38], confirming the high vulnerability of this region. Because of this, it cannot be excluded that the selective death of MNs (and general neuronal sufferance) occurring within the spinal cord of ALS patients might at least partially contribute to the dramatic loss of synaptic contacts here described. Still, our data indicate that all cell types within the spinal cord are currently identifiable by proteomics and, most important, are present in our *post-mortem* samples. This strengthens our theory that the synaptic changes we identified in this study are mainly linked to molecular alterations within remaining synapses, rather than neuronal loss. In addition, the integration of the *post-mortem* data with those obtained with hiPSC-derived MNs does not only represent an innovative combinatorial strategy for the investigation of synaptic features but also reduces the likelihood that the major findings hereby presented might arise from the reduced number of MNs in *post mortem* specimens. This also confirms hiPSCs as a *bona fide* model to investigate disease-related phenotypes and identify novel disease targets. Indeed, despite hiPSC-derived MNs resembling a premature synaptic transcriptome [74], our integrative strategy highlighted the synaptic vesicle release machinery as a commonly shared pathomechanism across our heterogeneous cohort of fALS cases.

Recent studies have also analyzed mutation-specific impacts on the cellular proteome. Since these analyses focused on the whole cell protein content, changes in small subcellular compartments such as the synapse are likely to be heavily underestimated, making the comparisons with the data hereby presented of difficult interpretation. However, despite this technical consideration, work from the Pasterkamp group described mutation-specific and convergent proteomic changes across hIPSC-derived MNs with different ALS-associated mutations [79]. Interestingly, this manuscript highlighted a significant enrichment of proteins associated with the presynaptic vesicle cycle in several analyses and a general convergence on mitochondrial dysfunction, in alignment with our findings. Similarly, Garone and colleagues [25] analyzed the whole proteome of FUS^P525L^ mutant iPSC-derived MNs and described a downregulation of processes such as “neuronal development”, “cytoskeleton organization” and several terms related to neuronal projection and axon guidance. Interestingly, cellular component analysis also showed enrichment of “vesicle” and “membrane-bound vesicle”, indicating potential convergence with our findings highlighting presynaptic vesicular dysfunction. Furthermore, Li and coworkers [41] used a multi-omics approach to study iPSC-derived MNs from *C9orf72* patients. Using whole cell homogenates, they described genomic, transcriptomic and proteomic changes within these cells, and looked for convergent changes across these techniques, revealing convergence on changes in the extracellular matrix and RNA processing. In line with other aspects of their work and other previous studies, we also found downregulated terms of lysosomal and mitochondrial processes in our *C9orf72* mutant lines [27, 44], and our post-mortem tissue also showed similar changes regardless of the genetic background. Analyzing the *TARDBP* mutation-specific changes, we found downregulation of cellular respiration in coherence with other patient-derived cell studies [73]. Interestingly, this study revealed that *TARDBP*-mutant iPSC-derived MNs differed in their transcriptomic profile depending on their mutation, however, they all shared common phenotypes of mitochondrial and synaptic dysfunction. Thus, despite different underlying genetic causes and presentation of TDP43 pathology (as in the case of SOD1 and FUS), it appears that common synaptic alterations do not represent a random event but rather a key pathological feature shared across the ALS spectrum at the proteomic, but also transcriptomic and even epigenetic level [13].

This synaptic convergence might be partially explained by the synaptic localization of C9orf72, FUS and TDP-43, which have been shown to play a local role in RNA [64, 84] and protein regulation [6, 86]. Of note, the levels of these 3 major ALS proteins, as well as of SOD1, were significantly higher in the synaptoneurosomes of ALS patients than in healthy controls. Moreover, altered presynaptic inputs to MNs have been observed upon accumulation of mutant SOD1 [32], indicating that the presence of the aggregation-prone form of the protein product of ALS genes might represent a common trigger of synaptic alterations and contribute to the accumulation of regulatory proteins due to impaired catabolism [14, 70]. This evidence might even support the spreading theory of cytotoxic protein species upon accumulation and failed clearance at the presynaptic site. Accordingly, OPTN, SQSTM1 and TBK1 were also increased in the ALS-related synaptic fractions, indicating that the autophagic alterations characterizing this disease [13, 48] might also occur locally at the synapse, representing a link between the accumulation of toxic aggregates and synaptic dysfunction. Indeed, our *post-mortem* samples were obtained from patients with TDP-43 pathology and we could identify several targets of this ALS-related gene, such as UNC13A [10], within the mutant synaptome. Moreover, the convergent SNARE phenotype observed in our models was also reproduced by overexpressing toxic poly(GA) aggregates, whose accumulation has been linked to presynaptic dysfunction and rescued by overexpressing the synaptic vesicle-related protein SV2 [31]. Of note, an altered SNARE complex has been also observed in Parkinson's (PD) and Alzheimer's disease (AD) [24, 71], where the cerebrospinal fluid levels of SNAP25 have been suggested to be representative of the progressive loss of synapse preceding neuronal loss [88]. Interestingly, SNARE and vesicles dynamics are also controlled by the c-Jun N-terminal kinase (JNK, aka MAPK8), which was the top candidate kinase associated with increased phospho-c-Jun^Ser63^ in ALS and whose activation has been associated with several stress conditions and neurodegenerative diseases [68, 85]. JNK interacts with some members of the SNARE complex such as SNAP25 at the presynapse [7] and inhibits the trafficking of synaptic vesicles upon nitric oxide-mediated stress [76]. In addition, we show that blocking the exocytosis of synaptic vesicles with TeNT is sufficient to enhance the levels of phospho-c-Jun, in agreement with the evidence of reduced activity being a key feature of vulnerable MNs in ALS [45]. This provides a possible mechanistic link, though speculative, between the altered machinery of vesicle release and the (mal)activation of c-Jun at Serine 63, which we identified as a common phenotype in the presence of different ALS mutations. Given the extreme genetic heterogeneity of this disease, our evidence of a commonly shared alteration represents a novel and potentially widely impactful therapeutic opportunity. Here, we tested this hypothesis by taking advantage of patient-derived MNs to demonstrate that this might indeed represent an efficient strategy in a large spectrum of ALS cases. In fact, DHA treatment did not only elevate the SYP levels in mutant cultures (as well as Healthy ones), but it also exerted a neuroprotective effect by improving cell survival and reducing the phospho-c-Jun levels in ALS MNs, supporting the evidences that resolving synaptic impairments can efficiently contrast neuronal degeneration [4, 14, 65]. Despite the biological interconnection between DHA, which is the most abundant PUFA in the CNS [37], and synaptic proteins still has to be completely elucidated, this fatty acid was proven neuroprotective in different models of neurodegeneration and brain injury. DHA indeed rescues neuronal degeneration in AD neurons [35] and ameliorates the conditions of rats after traumatic brain injury [92]. In addition, DHA reduces the formation of brain edema after injury by stabilizing the function of the blood–brain barrier [42], whose disruption has been recently shown to represent a common feature of different ALS murine models [2]. Mechanistically, it has been shown that DHA can even modulate gene expression [52] by activating specific transcription factors such as CREB [90], whose reduced function has been linked to the loss of synaptic transcripts in ALS [14]. Accordingly, DHA was shown to stimulate synaptogenesis as well as SNARE-mediated docking of synaptic vesicles [11, 89]. Since a diet rich in PUFAs has been suggested to reduce the risk of developing ALS [23], our data provide important insights into potentially relevant biochemical mechanisms that might explain the neuroprotective effect of DHA.

In conclusion, this study provides the first in-depth analysis of the human spinal synaptic proteome, which was found to be dramatically altered in ALS. The observed synaptic alterations were strongly conserved in sporadic and *C9orf72*-fALS patients and could be reliably reproduced in a heterogeneous panel of ALS hiPSC-derived MNs, revealing the synaptic microenvironment as a valid target for the future development of novel therapeutic strategies against MNs degeneration.

### Limitations of the study

While our work highlights important convergent mechanisms across a range of human samples with different ALS genetic backgrounds, there are inherent limitations in our utilization of *post-mortem* samples, which we decided to pool before performing mass-spectrometry. This allowed us to overcome the technical variability within the proteomic analysis and only reveal changes that were convergent across our samples within the pool. However, this approach prevents us from analyzing any association between an individual’s clinical presentation and disease course, with alterations in their synaptic proteome. It makes sense that a longer disease course may result in more severe synaptic alterations, yet this level of analytical complexity and insight is lost by pooling individual samples. Furthermore, this approach prevents our ability to align synaptic alterations with the individual presence of neuropathology and its severity. For example, we discovered that several synaptic proteins whose levels are altered in ALS are also potentially under the regulatory control of TDP-43. It would be interesting to assess, at the individual level, whether the expression levels of these proteins were more severely altered in patients with more severe pTDP-43 neuropathology.

Lastly, the hiPSC lines used in this study covered a large portion of the familial ALS spectrum, but these were not originated from the same patients we obtained *post-mortem* samples from and we also did not have access to sporadic ones to fully match the in vivo and in vitro datasets.

## Supplementary Information

Below is the link to the electronic supplementary material.

Supplementary file1 (PDF 5871 kb)

Supplementary file2 (XLSX 11 kb)

Supplementary file3 (XLSX 3303 kb)

Supplementary file4 (XLSX 253 kb)

## Data Availability

The datasets generated and/or analysed during the current study are available in the PRIDE repository and are available with the dataset identifiers PXD041543 (hiPSC-derivewd MNs) and PXD042617 (*post mortem samples*).
