# Correction to: Integrative proteomics highlight presynaptic alterations and c-Jun misactivation as convergent pathomechanisms in ALS

**DOI:** 10.1007/s00401-023-02630-9

**Published:** 2023-09-12

**Authors:** Amr Aly, Zsofia I. Laszlo, Sandeep Rajkumar, Tugba Demir, Nicole Hindley, Douglas J. Lamont, Johannes Lehmann, Mira Seidel, Daniel Sommer, Mirita Franz-Wachtel, Francesca Barletta, Simon Heumos, Stefan Czemmel, Edor Kabashi, Albert Ludolph, Tobias M. Boeckers, Christopher M. Henstridge, Alberto Catanese

**Affiliations:** 1 Institute of Anatomy and Cell Biology, Ulm University, Ulm, Germany; 2 Division of Cellular and Systems Medicine, School of Medicine, University of Dundee, Dundee, Scotland, UK; 3 FingerPrints Proteomics Facility, Discovery Centre, School of Life Sciences, University of Dundee, Dundee, Scotland, UK; 4 Proteome Center Tübingen, University of Tübingen, 72076 Tübingen, Germany; 5 Quantitative Biology Center (QBiC), University of Tübingen, 72076 Tübingen, Germany; 6 Biomedical Data Science, Department of Computer Science, University of Tübingen, 72076 Tübingen, Germany; 7 Laboratory of Translational Research for Neurological Disorders, Imagine Institute, Université de Paris, INSERM, UMR 1163, 75015 Paris, France; 8 Department of Neurology, Ulm University School of Medicine, Ulm, Germany; 9 German Center for Neurodegenerative Diseases (DZNE), Ulm Site, Germany

**Correction to: Acta Neuropathologica (2023) 146:451–475** 10.1007/s00401-023-02611-y

The wrong Supplementary files (Figure and Table 2) were originally published with this article; it has now been replaced with the correct file.

The original article has been corrected.

### Supplementary Information

Below is the link to the electronic supplementary material.

Supplementary file1 (PDF 5870 KB)

Supplementary file2 (XLSX 3302 KB)

## Data Availability

The MS proteomics data have been deposited to the ProteomeXchange Consortium (http://proteomecentral.proteomexchange.org) via the PRIDE partner repository and are available with the dataset identifiers PXD041543 (hiPSC-derivewd MNs) and PXD042617 (post mortem samples).

