# Supplementary material for: Integrative proteomics highlight presynaptic alterations and c-Jun misactivation as convergent pathomechanisms in ALS
Source: Acta Neuropathol. 2023 Jul 24;146(3):451–75. doi: 10.1007/s00401-023-02611-y (PMC10412488; doi:10.1007/s00401-023-02611-y)
Supplement: Supplementary file 1 — Supplementary file1 (PDF 5871 kb) [file 401_2023_2611_MOESM1_ESM.pdf]

Integrative proteomics highlight presynaptic alterations and c-Jun misactivation as convergent pathomechanisms in ALS

Amr Aly[1,*], Zsofia I. Laszlo[2,*], Sandeep Rajkumar[1], Tugba Demir[1], Nicole Hindley[2], Douglas J. Lamont[3], Johannes Lehmann[1], Mira Seidel[1], Daniel Sommer[1], Mirita Franz-Wachtel[4], Francesca Barletta[5], Simon Heumos[5,6], Stefan Czemmel[5], Edor Kabashi[7], Albert Ludolph[8,9], Tobias M. Boeckers[1,9], Christopher M. Henstridge[2,†], Alberto Catanese[1,9,†]

1 Institute of Anatomy and Cell Biology, Ulm University, Ulm, Germany
2 Division of Cellular and Systems Medicine, School of Medicine, University of Dundee, Dundee, Scotland, UK
3 FingerPrints Proteomics Facility, Discovery Centre, School of Life Sciences, University of Dundee, Dundee, Scotland, UK
4 Proteome Center Tübingen, University of Tübingen, 72076, Tübingen, Germany.
5 Quantitative Biology Center (QBiC), University of Tübingen, 72076, Tübingen, Germany.
6 Biomedical Data Science, Dept. of Computer Science, University of Tübingen, 72076, Tübingen, Germany
7 Laboratory of Translational Research for Neurological Disorders, Imagine Institute, Université de Paris, INSERM UMR 1163, 75015, Paris, France.
8 Department of Neurology, Ulm University School of Medicine, Ulm, Germany
9 German Center for Neurodegenerative Diseases (DZNE), Ulm site

* co-first author
† co-corresponding author

Correspondence to:
Dr. Christopher Henstridge
Email: c.henstridge@dundee.ac.uk
Tel: +44 (0)1382 388676

Dr. Alberto Catanese
Email: alberto.catanese@uni-ulm.de
Tel: +49 (0)731 50023213

Supplementary Figures 1-25

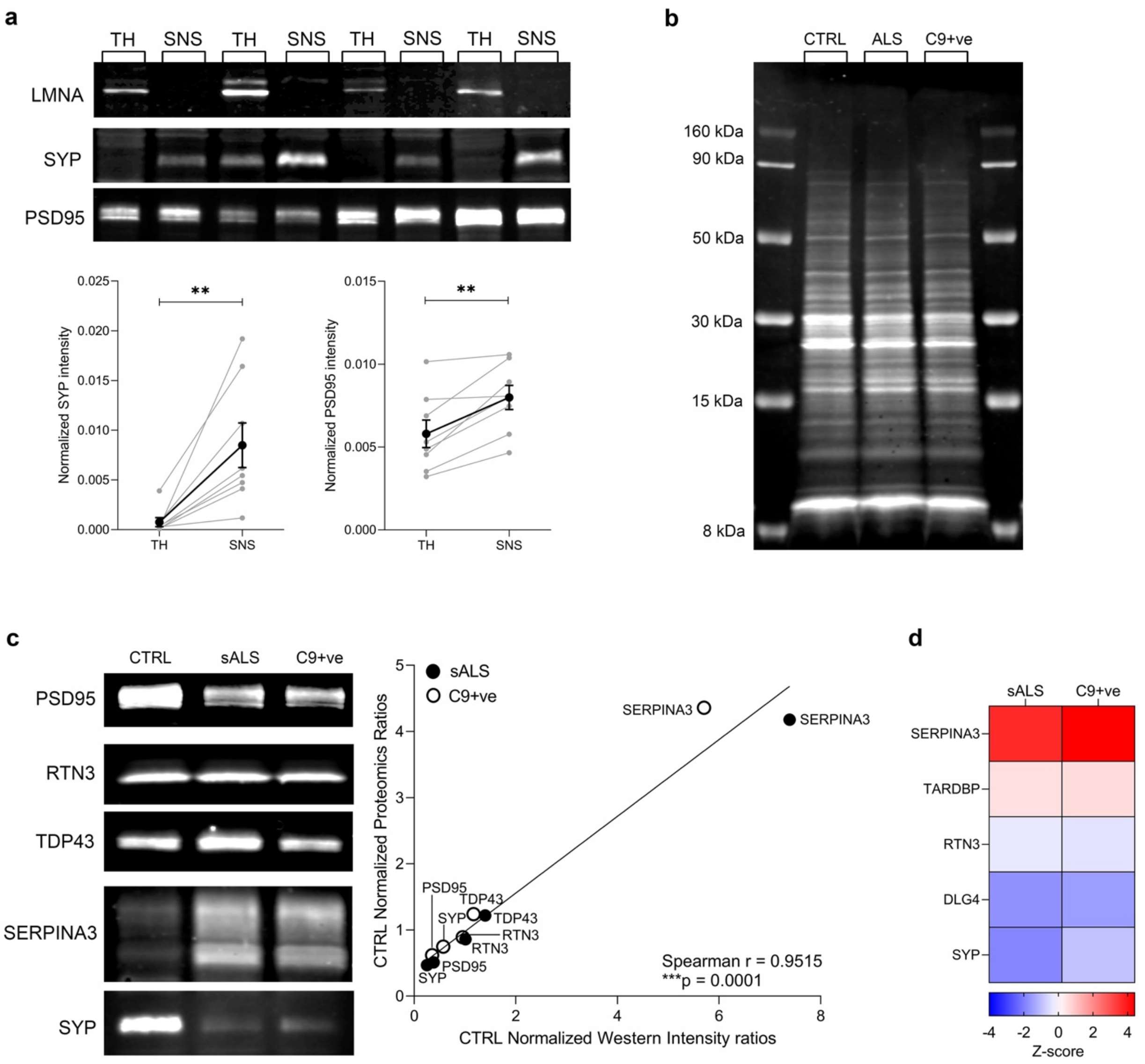

Supplementary Fig. 1 Western blot validation of synaptically-enriched fractions from *post-mortem* samples. **a** Western blot from control samples confirm exclusion of nuclear protein (lamin) and enrichment of synaptic proteins (SYP and PSD95) in synaptoneurosome (SNS) compared to total homogenate (TH) fractions. Graphs show mean±SEM analysed by Paired t-test in case of PSD95 **p=0.0036 and Wilcoxon test in case of SYP **p=0-0078. **b** Total protein stain demonstrate the quality of samples used for further proteomics analysis. **c** Representative western blot images using the pooled samples shows significant correlation with proteomics results. Control normalized western blot intensity values were plotted against proteomics control normalized ratiometric values. Spearman correlation ***p=0.0001, r=0.9515. **d** Heatmap representation of the used proteins

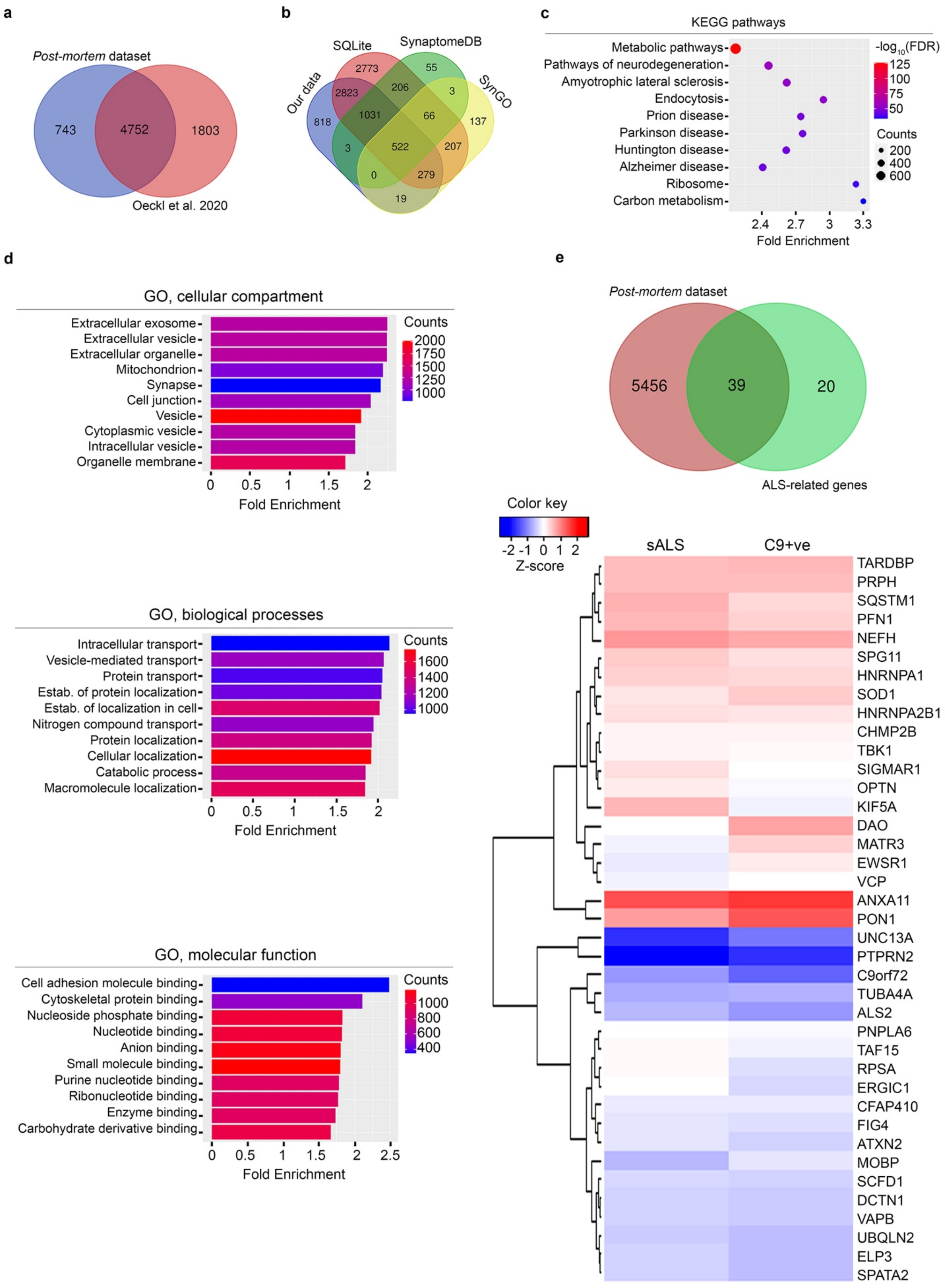
a
Post-mortem dataset
743
4752
1803
Oeckl et al. 2020
b
SQLite
SynaptomeDB
Our data
SynGO
2773
55
2823
206
3
818
1031
66
137
3
522
207
0
279
19
c
KEGG pathways
Metabolic pathways
Pathways of neurodegeneration
Amyotrophic lateral sclerosis
Endocytosis
Prion disease
Parkinson disease
Huntington disease
Alzheimer disease
Ribosome
Carbon metabolism
2.4 2.7 3 3.3
Fold Enrichment
-log10(FDR)
125 100 75 50
Counts
200 400 600
d
GO, cellular compartment
Extracellular exosome
Extracellular vesicle
Extracellular organelle
Mitochondrion
Synapse
Cell junction
Vesicle
Cytoplasmic vesicle
Intracellular vesicle
Organelle membrane
0 0.5 1 1.5 2
Fold Enrichment
Counts
2000 1750 1500 1250 1000
GO, biological processes
Intracellular transport
Vesicle-mediated transport
Protein transport
Estab. of protein localization
Estab. of localization in cell
Nitrogen compound transport
Protein localization
Cellular localization
Catabolic process
Macromolecule localization
0 0.5 1 1.5 2
Fold Enrichment
Counts
1600 1400 1200 1000
GO, molecular function
Cell adhesion molecule binding
Cytoskeletal protein binding
Nucleoside phosphate binding
Nucleotide binding
Anion binding
Small molecule binding
Purine nucleotide binding
Ribonucleotide binding
Enzyme binding
Carbohydrate derivative binding
0 0.5 1 1.5 2 2.5
Fold Enrichment
Counts
1000 800 600 400
e
Post-mortem dataset
5456
39
20
ALS-related genes
Color key
-2 -1 0 1 2
Z-score
sALS
C9+ve
TARDBP
PRPH
SQSTM1
PFN1
NEFH
SPG11
HNRNPA1
SOD1
HNRNPA2B1
CHMP2B
TBK1
SIGMAR1
OPTN
KIF5A
DAO
MATR3
EWSR1
VCP
ANXA11
PON1
UNC13A
PTPRN2
C9orf72
TUBA4A
ALS2
PNPLA6
TAF15
RPSA
ERGIC1
CFAP410
FIG4
ATXN2
MOBP
SCFD1
DCTN1
VAPB
UBQLN2
ELP3
SPATA2

Supplementary Fig. 2 Bioinformatics validation of the *post-mortem* synaptic proteome. **a** The identified 5495 unique ID shows 86% overlap with previously published human spinal cord dataset. **b** 85% of our dataset provide alignment with different synaptic databases validating the composition of the synaptic fractions. **c-d** Enrichment analysis was performed using the overall dataset, KEGG pathways and Gene Ontology (GO) terms were plotted using the top 10 terms plotted by fold enrichment, $-\log_{10}$(FDR) (**c**) and gene count (**d**). **e** Venn diagram displays 39 ALS-related gene products were found in our synaptically-enriched fraction. The expression of the 39 ALS-specific genes were visualized by heatmap using hierarchical clustering with Euclidean distance

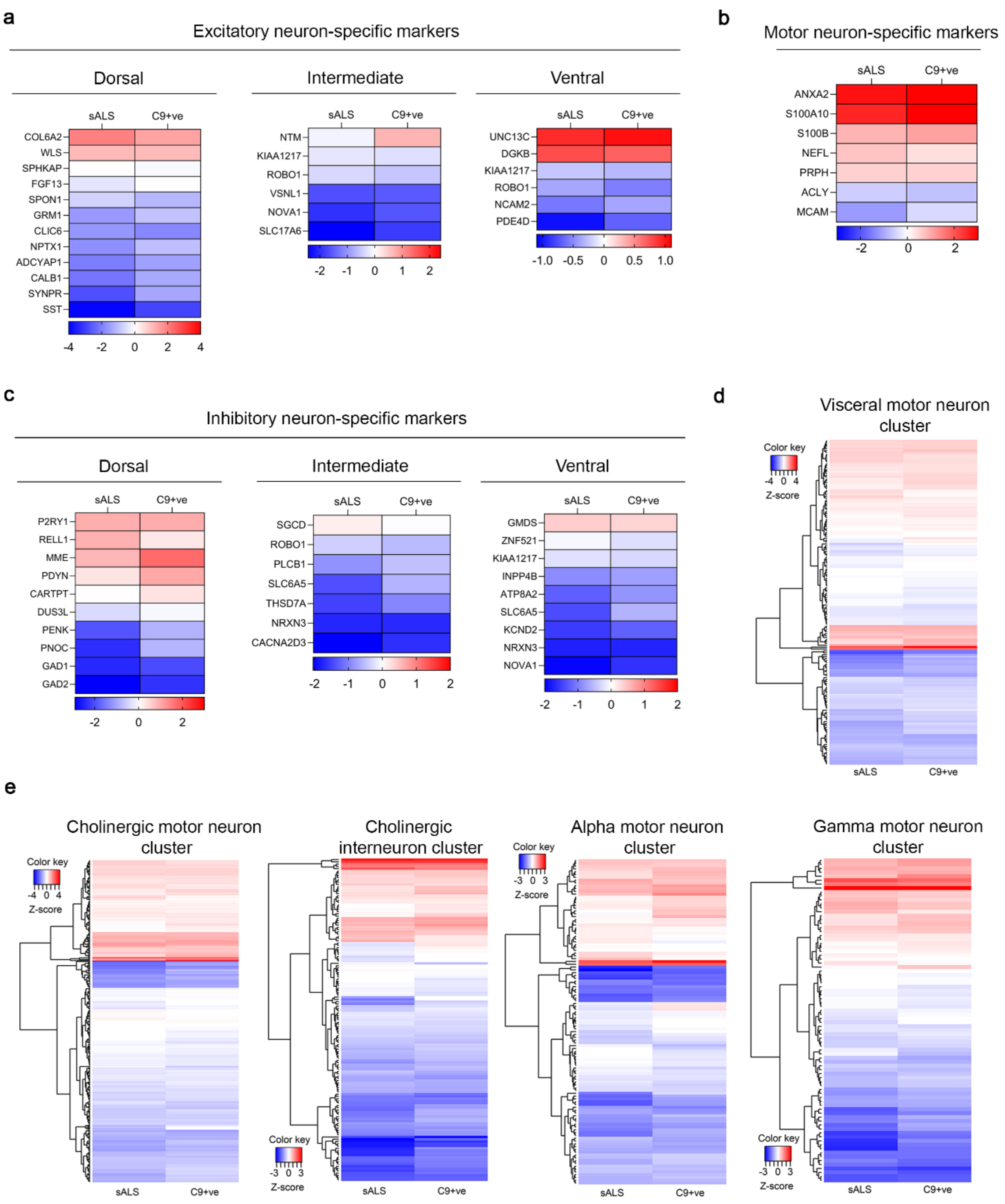

Supplementary Fig. 3 Cell type-specific enrichment analysis of the *post-mortem* dataset. **a-c** Region- and cell type-specific protein clusters were plotted using the cellular taxonomy of the human spinal cord. **d-e** Motor neuron subtype-specific clusters were initially identified by Blum *et al*. 2021 [8] and aligned with our synaptic *post-mortem* dataset. Heatmap was created using hierarchical clustering with Euclidean distance.

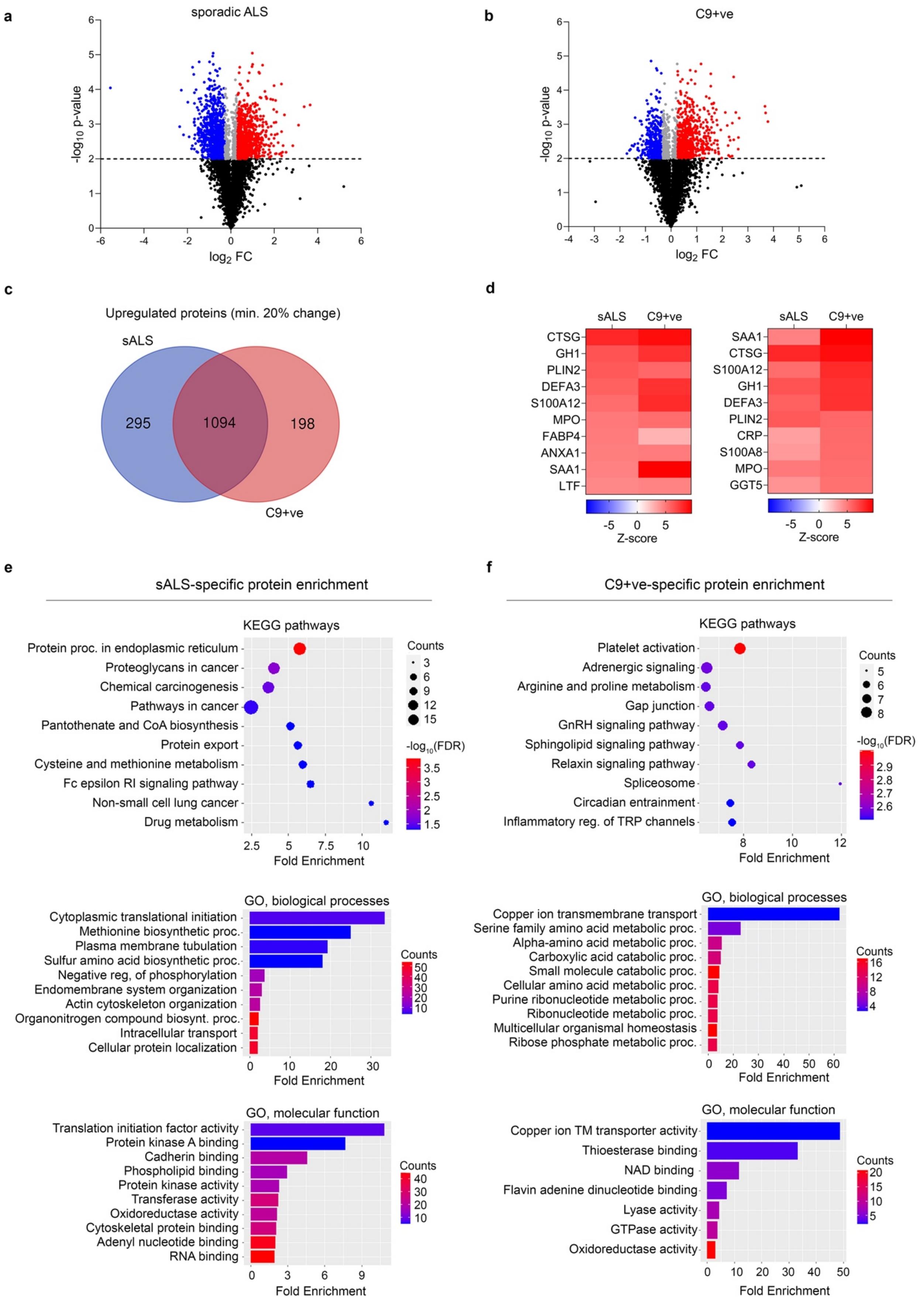

a
sporadic ALS
-log10 p-value
log2 FC
b
C9+ve
-log10 p-value
log2 FC
c
Upregulated proteins (min. 20% change)
sALS
295
1094
198
C9+ve
d
sALS
C9+ve
CTSG
GH1
PLIN2
DEFA3
S100A12
MPO
FABP4
ANXA1
SAA1
LTF
SAA1
CTSG
S100A12
GH1
DEFA3
PLIN2
CRP
S100A8
MPO
GGT5
Z-score
e
sALS-specific protein enrichment
KEGG pathways
Protein proc. in endoplasmic reticulum
Proteoglycans in cancer
Chemical carcinogenesis
Pathways in cancer
Pantothenate and CoA biosynthesis
Protein export
Cysteine and methionine metabolism
Fc epsilon RI signaling pathway
Non-small cell lung cancer
Drug metabolism
Counts
-log10(FDR)
Fold Enrichment
GO, biological processes
Cytoplasmic translational initiation
Methionine biosynthetic proc.
Plasma membrane tubulation
Sulfur amino acid biosynthetic proc.
Negative reg, of phosphorylation
Endomembrane system organization
Actin cytoskeleton organization
Organonitrogen compound biosynt. proc.
Intracellular transport
Cellular protein localization
Counts
Fold Enrichment
GO, molecular function
Translation initiation factor activity
Protein kinase A binding
Cadherin binding
Phospholipid binding
Protein kinase activity
Transferase activity
Oxidoreductase activity
Cytoskeletal protein binding
Adenyl nucleotide binding
RNA binding
Counts
Fold Enrichment
f
C9+ve-specific protein enrichment
KEGG pathways
Platelet activation
Adrenergic signaling
Arginine and proline metabolism
Gap junction
GnRH signaling pathway
Sphingolipid signaling pathway
Relaxin signaling pathway
Spliceosome
Circadian entrainment
Inflammatory reg. of TRP channels
Counts
-log10(FDR)
Fold Enrichment
GO, biological processes
Copper ion transmembrane transport
Serine family amino acid metabolic proc.
Alpha-amino acid metabolic proc.
Carboxylic acid catabolic proc.
Small molecule catabolic proc.
Cellular amino acid metabolic proc.
Purine ribonucleotide metabolic proc.
Ribonucleotide metabolic proc.
Multicellular organismal homeostasis
Ribose phosphate metabolic proc.
Counts
Fold Enrichment
GO, molecular function
Copper ion TM transporter activity
Thioesterase binding
NAD binding
Flavin adenine dinucleotide binding
Lyase activity
GTPase activity
Oxidoreductase activity
Counts
Fold Enrichment

Supplementary Fig. 4 ALS subtype-specific enrichment analysis of the upregulated proteins. **a-b** Volcano plots represent the protein distribution in sALS (**a**) and C9+ve samples (**b**) using $\log_2$ fold change and $-\log_{10}$ p value per protein. Significant changes were determined using 2≤ threshold for $-\log_{10}$ p value and 0.3≤ for upregulated and -0.3≥ for downregulated proteins. **c** Venn diagram shows the number of ALS-subtype specific upregulated proteins. **d** Top 10 upregulated proteins specifically changed in the sporadic and C9+ve groups. **e** sALS-specific enrichment analysis shows the top 10 KEGG pathways and Gene Ontology terms. **f** C9+ve-specific upregulated proteins were used to perform enrichment analysis. Plots were created and colorized based on fold enrichment, gene count per pathway and $-\log_{10}$(FDR).

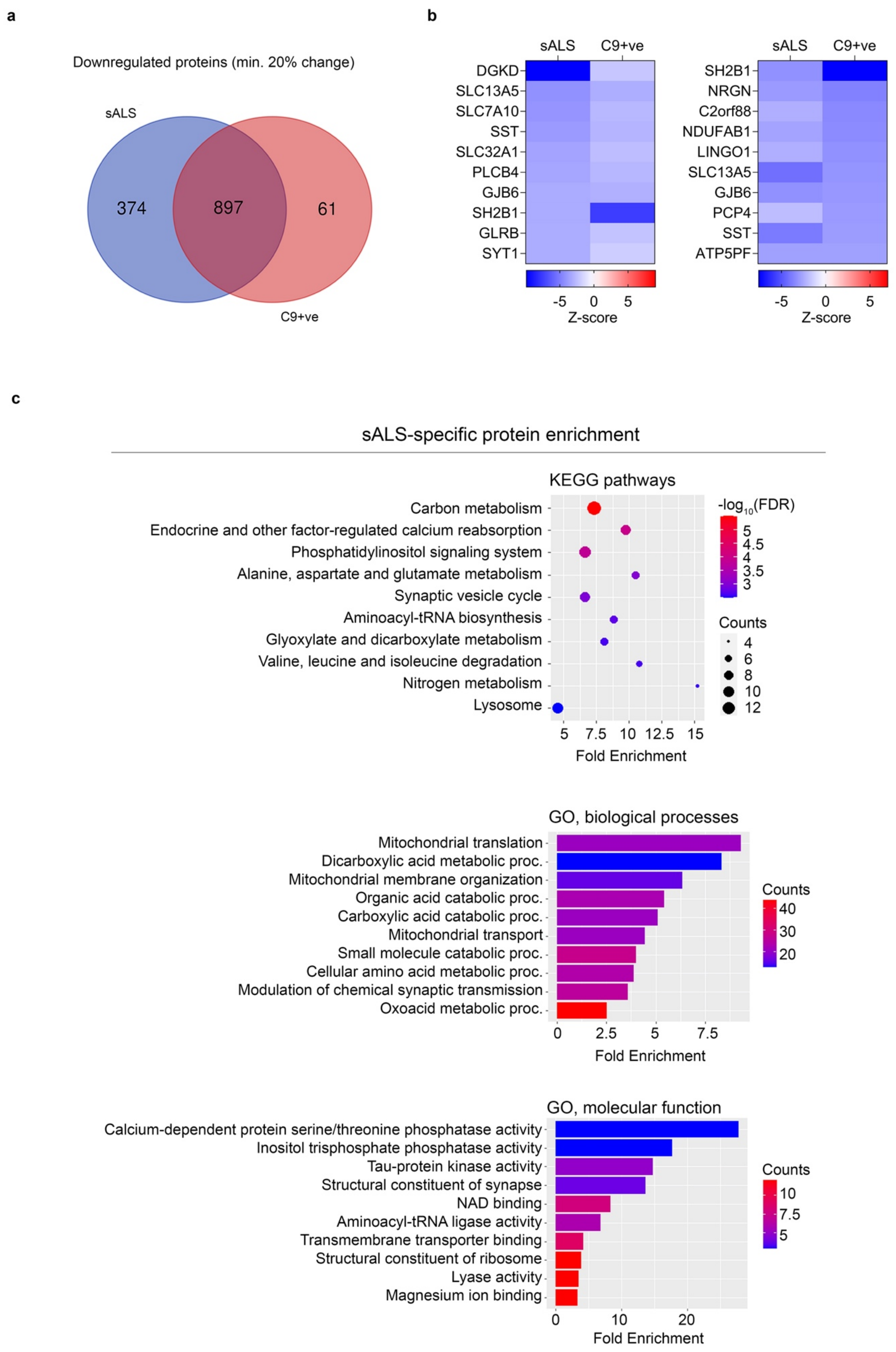
a
Downregulated proteins (min. 20% change)
sALS
374
897
61
C9+ve
b
sALS
C9+ve
DGKD
SLC13A5
SLC7A10
SST
SLC32A1
PLCB4
GJB6
SH2B1
GLRB
SYT1
-5
0
5
Z-score
sALS
C9+ve
SH2B1
NRGN
C2orf88
NDUFAB1
LINGO1
SLC13A5
GJB6
PCP4
SST
ATP5PF
-5
0
5
Z-score
c
sALS-specific protein enrichment
KEGG pathways
Carbon metabolism
Endocrine and other factor-regulated calcium reabsorption
Phosphatidylinositol signaling system
Alanine, aspartate and glutamate metabolism
Synaptic vesicle cycle
Aminoacyl-tRNA biosynthesis
Glyoxylate and dicarboxylate metabolism
Valine, leucine and isoleucine degradation
Nitrogen metabolism
Lysosome
5
7.5
10
12.5
15
Fold Enrichment
$-\log_{10}$(FDR)
5
4.5
4
3.5
3
Counts
4
6
8
10
12
GO, biological processes
Mitochondrial translation
Dicarboxylic acid metabolic proc.
Mitochondrial membrane organization
Organic acid catabolic proc.
Carboxylic acid catabolic proc.
Mitochondrial transport
Small molecule catabolic proc.
Cellular amino acid metabolic proc.
Modulation of chemical synaptic transmission
Oxoacid metabolic proc.
0
2.5
5
7.5
Fold Enrichment
Counts
40
30
20
GO, molecular function
Calcium-dependent protein serine/threonine phosphatase activity
Inositol trisphosphate phosphatase activity
Tau-protein kinase activity
Structural constituent of synapse
NAD binding
Aminoacyl-tRNA ligase activity
Transmembrane transporter binding
Structural constituent of ribosome
Lyase activity
Magnesium ion binding
0
10
20
Fold Enrichment
Counts
10
7.5
5

Supplementary Fig. 5 ALS subtype-specific enrichment analysis of the downregulated proteins. **a** Venn diagram shows the alignment of the downregulated protein clusters from both ALS groups. **b** Subtype-specific top 10 downregulated proteins visualized by heatmap. **c** sALS-specific downregulated proteins were used to perform enrichment analysis. The top 10 KEGG pathways and Gene Ontology (GO) terms were plotted and colorized based on fold enrichment, gene count per pathway and $-\log_{10}$(FDR). The C9+ve-specific downregulated protein cluster (61) did not show any kind of specific term enrichment

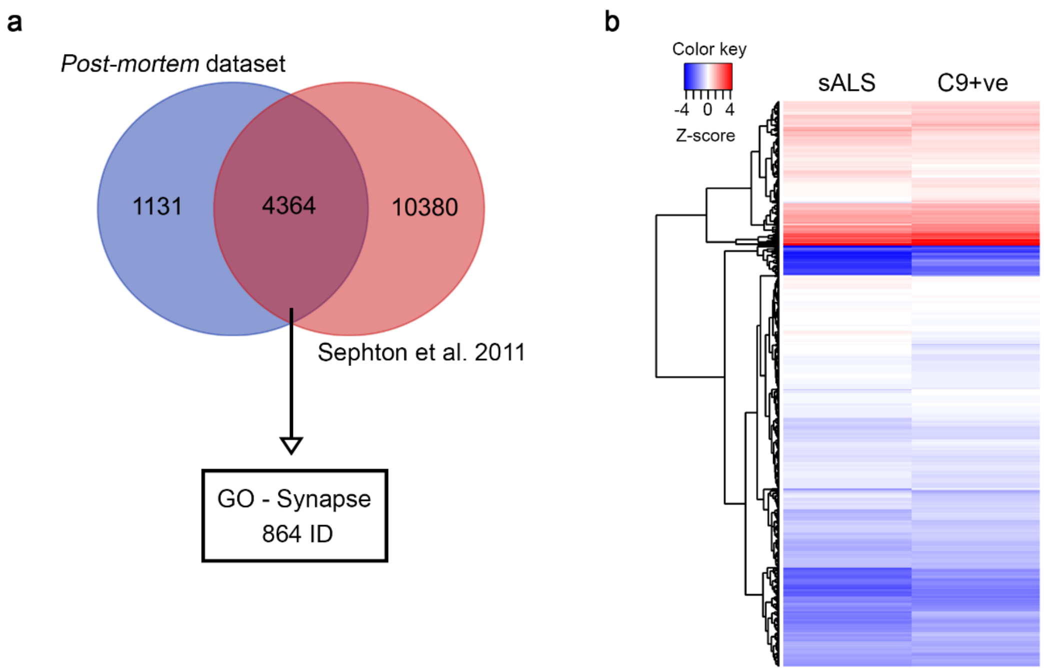

Supplementary Fig. 6 Selection of synaptic TDP43 interactors. **a** Venn diagram displays the alignment of the *post-mortem* dataset with the TDP43 interactome from the Sephton *et al*. 2011 study [69]. Based on the Gene Ontology term „Synapse" 864 protein IDs were identified as synaptic proteins. **b** Expression of the synaptic interactors of TDP43 was plotted using hierarchical clustering with Euclidean distance.

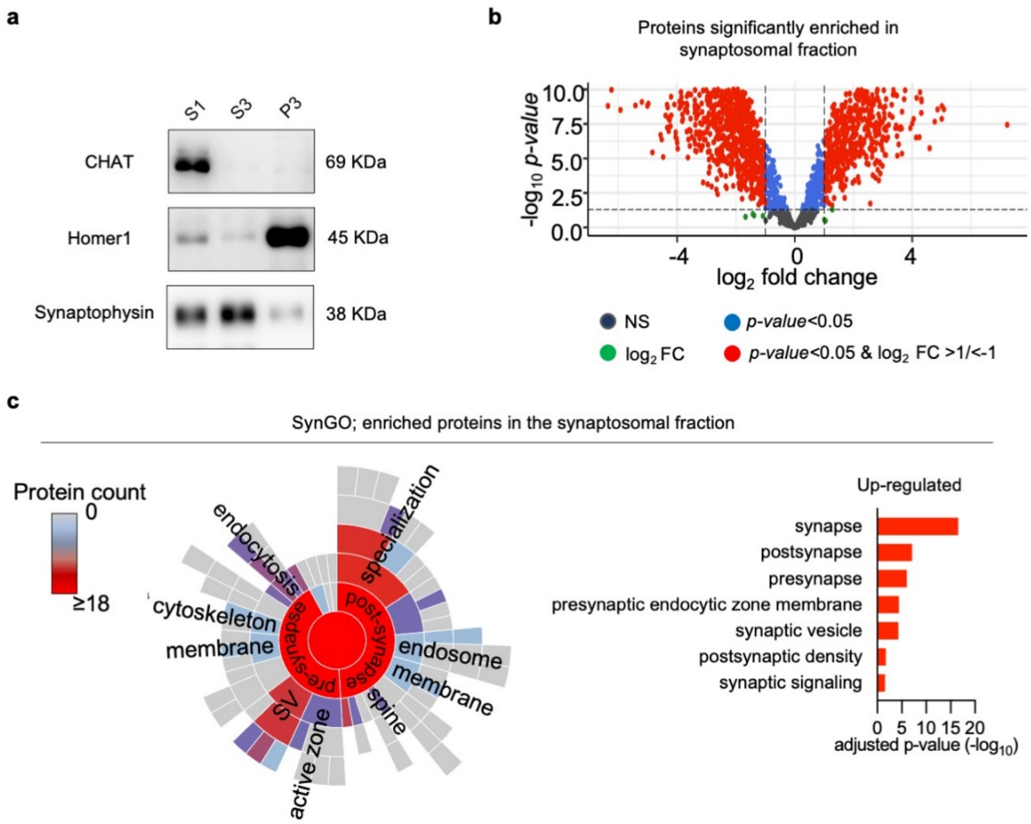

Supplementary Fig. 7 Validation of synaptic enrichment after fractionation of hiPSC-derived MN samples. **a** Western blot analysis confirms the enrichment of synaptic proteins in the synaptic cytosol (S3) and post-synaptic density (P3) fractions. **b** Volcano plots represent the protein distribution in the synaptosomal fraction and the total extra-nuclear lysate (P1) confirming that the synaptic fractions were indeed significantly enriched in proteins belonging to this neuronal microenvironment. **c** Synaptic enrichment analysis was confirmed using SynGO database, revealing enriched synaptic functional terms such as synaptic vesicle cycle, presynaptic endocytosis and post-synaptic density in the synaptosomal fraction

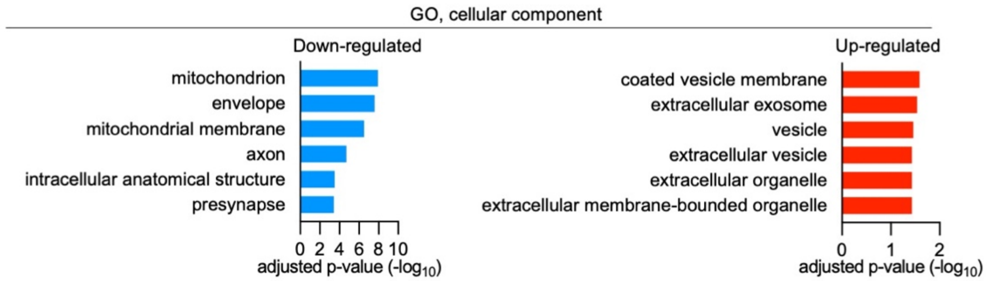

Supplementary Fig. 8 Enrichment analysis of synaptic proteome from hiPSC-derived MNs from ALS patients. Significantly down- and up-regulated GO (cellular component) terms in ALS based on the synaptome dataset

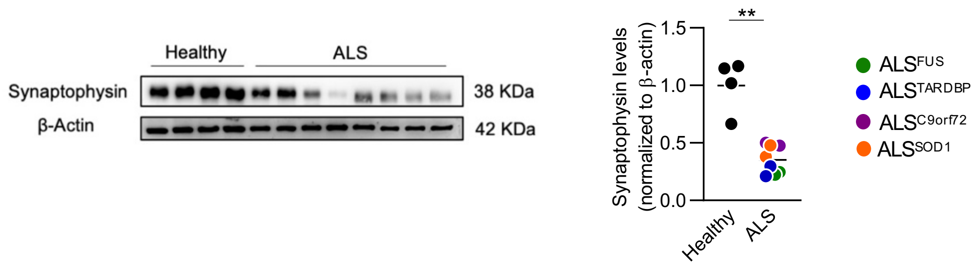

Supplementary Fig. 9 Confirmation of Synaptophysin down-regulation in hiPSC-derived ALS MNs**.** Western blot analysis of the presynaptic fraction revealed a significant reduction of SYP levels in all the mutant lines when compared to the four controls, confirming the synaptic proteome results. Welch's *t*-test $^{**}P < 0.01$. $n = 3$ independent cultures

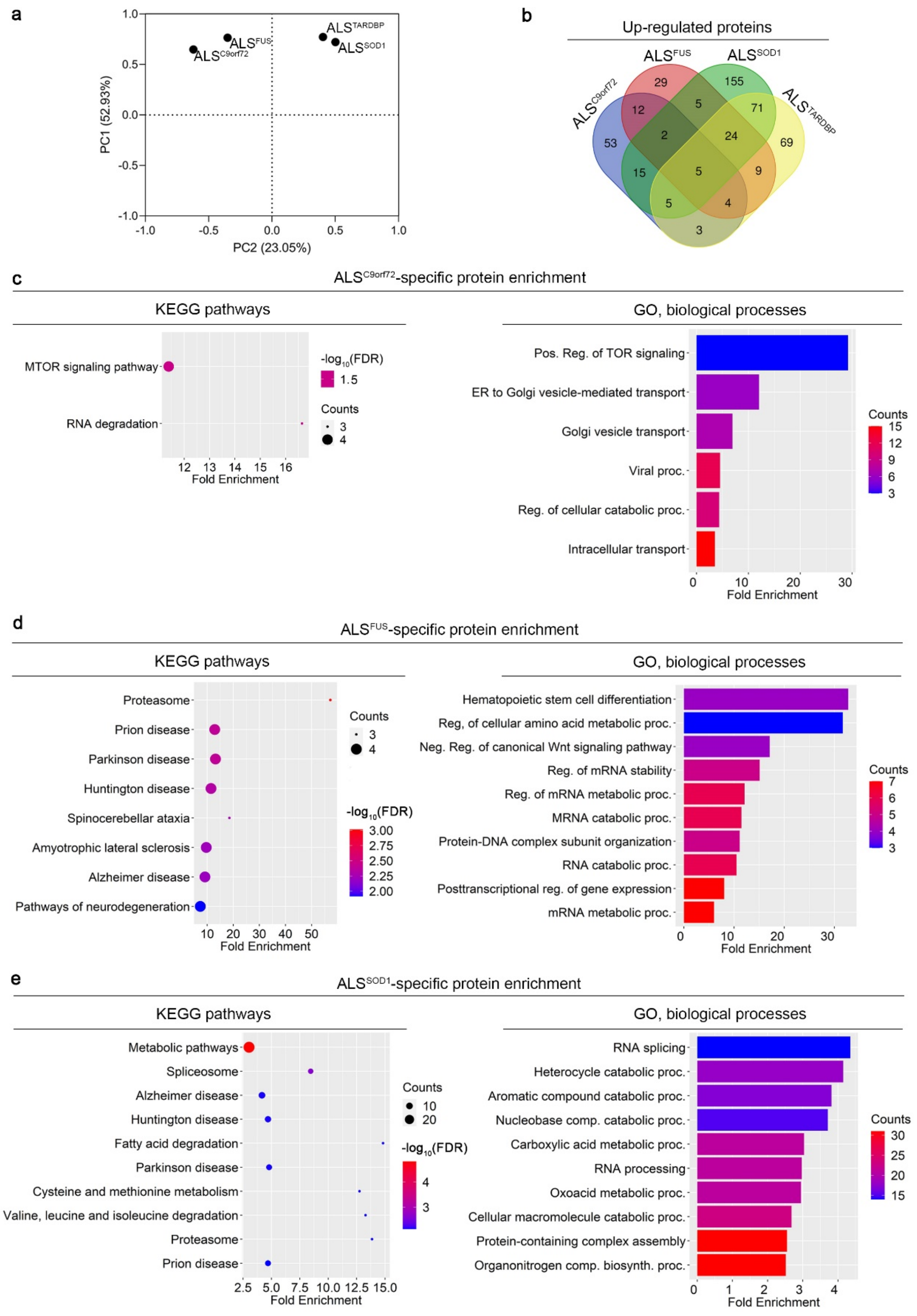

a
PC1 (52.93%)
PC2 (23.05%)
ALS^{TARDBP}
ALS^{FUS}
ALS^{SOD1}
$ALS^{C9orf72}$
b
Up-regulated proteins
ALS^{FUS}
ALS^{SOD1}
$ALS^{C9orf72}$
ALS^{TARDBP}
c
$ALS^{C9orf72}$-specific protein enrichment
KEGG pathways
MTOR signaling pathway
RNA degradation
$-log_{10}(FDR)$
Counts
Fold Enrichment
GO, biological processes
Pos. Reg. of TOR signaling
ER to Golgi vesicle-mediated transport
Golgi vesicle transport
Viral proc.
Reg. of cellular catabolic proc.
Intracellular transport
Counts
Fold Enrichment
d
ALS^{FUS}-specific protein enrichment
KEGG pathways
Proteasome
Prion disease
Parkinson disease
Huntington disease
Spinocerebellar ataxia
Amyotrophic lateral sclerosis
Alzheimer disease
Pathways of neurodegeneration
Counts
$-log_{10}(FDR)$
Fold Enrichment
GO, biological processes
Hematopoietic stem cell differentiation
Reg, of cellular amino acid metabolic proc.
Neg. Reg. of canonical Wnt signaling pathway
Reg. of mRNA stability
Reg. of mRNA metabolic proc.
MRNA catabolic proc.
Protein-DNA complex subunit organization
RNA catabolic proc.
Posttranscriptional reg. of gene expression
mRNA metabolic proc.
Counts
Fold Enrichment
e
ALS^{SOD1}-specific protein enrichment
KEGG pathways
Metabolic pathways
Spliceosome
Alzheimer disease
Huntington disease
Fatty acid degradation
Parkinson disease
Cysteine and methionine metabolism
Valine, leucine and isoleucine degradation
Proteasome
Prion disease
Counts
$-log_{10}(FDR)$
Fold Enrichment
GO, biological processes
RNA splicing
Heterocycle catabolic proc.
Aromatic compound catabolic proc.
Nucleobase comp. catabolic proc.
Carboxylic acid metabolic proc.
RNA processing
Oxoacid metabolic proc.
Cellular macromolecule catabolic proc.
Protein-containing complex assembly
Organonitrogen comp. biosynth. proc.
Counts
Fold Enrichment

Supplementary Fig. 10 Enrichment analysis of the ALS subtype-specific up-regulated proteins. **a** Principal component analysis using the control normalized expression values shows the clustering of the different ALS MN cell lines. **b** Venn diagram visualizes the alignment of the mutation-specific up-regulated proteins. Enrichment analysis shows the top KEGG pathways and Gene Ontology (GO) terms using *C9orf72*- (**c**), *FUS*- (**d**) and *SOD1*-specific up-regulated protein IDs (**e**).

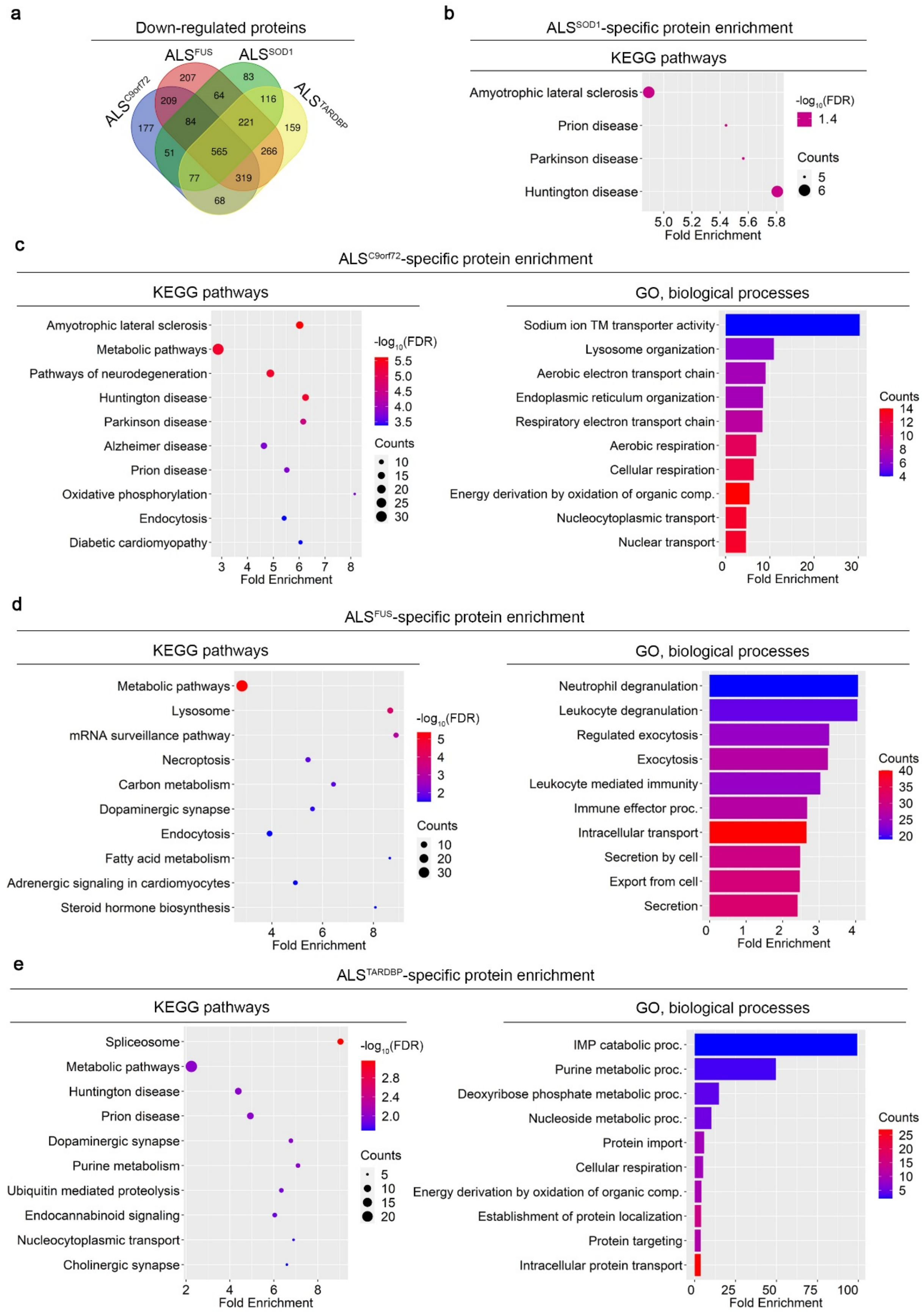
a
Down-regulated proteins
ALSFUS
ALSSOD1
ALSC9orf72
ALSTARDBP
207
83
209
64
116
177
84
221
159
51
565
266
77
319
68
b
ALSSOD1-specific protein enrichment
KEGG pathways
Amyotrophic lateral sclerosis
Prion disease
Parkinson disease
Huntington disease
-log10(FDR)
1.4
Counts
5
6
5.0 5.2 5.4 5.6 5.8
Fold Enrichment
c
ALSC9orf72-specific protein enrichment
KEGG pathways
Amyotrophic lateral sclerosis
Metabolic pathways
Pathways of neurodegeneration
Huntington disease
Parkinson disease
Alzheimer disease
Prion disease
Oxidative phosphorylation
Endocytosis
Diabetic cardiomyopathy
-log10(FDR)
5.5
5.0
4.5
4.0
3.5
Counts
10
15
20
25
30
3 4 5 6 7 8
Fold Enrichment
GO, biological processes
Sodium ion TM transporter activity
Lysosome organization
Aerobic electron transport chain
Endoplasmic reticulum organization
Respiratory electron transport chain
Aerobic respiration
Cellular respiration
Energy derivation by oxidation of organic comp.
Nucleocytoplasmic transport
Nuclear transport
Counts
14
12
10
8
6
4
0 10 20 30
Fold Enrichment
d
ALSFUS-specific protein enrichment
KEGG pathways
Metabolic pathways
Lysosome
mRNA surveillance pathway
Necroptosis
Carbon metabolism
Dopaminergic synapse
Endocytosis
Fatty acid metabolism
Adrenergic signaling in cardiomyocytes
Steroid hormone biosynthesis
-log10(FDR)
5
4
3
2
Counts
10
20
30
4 6 8
Fold Enrichment
GO, biological processes
Neutrophil degranulation
Leukocyte degranulation
Regulated exocytosis
Exocytosis
Leukocyte mediated immunity
Immune effector proc.
Intracellular transport
Secretion by cell
Export from cell
Secretion
Counts
40
35
30
25
20
0 1 2 3 4
Fold Enrichment
e
ALSTARDBP-specific protein enrichment
KEGG pathways
Spliceosome
Metabolic pathways
Huntington disease
Prion disease
Dopaminergic synapse
Purine metabolism
Ubiquitin mediated proteolysis
Endocannabinoid signaling
Nucleocytoplasmic transport
Cholinergic synapse
-log10(FDR)
2.8
2.4
2.0
Counts
5
10
15
20
2 4 6 8
Fold Enrichment
GO, biological processes
IMP catabolic proc.
Purine metabolic proc.
Deoxyribose phosphate metabolic proc.
Nucleoside metabolic proc.
Protein import
Cellular respiration
Energy derivation by oxidation of organic comp.
Establishment of protein localization
Protein targeting
Intracellular protein transport
Counts
25
20
15
10
5
0 25 50 75 100
Fold Enrichment

Supplementary Fig. 11 Alignment and enrichment analysis of the mutant cell line-specific down-regulated proteins. **a** Venn diagram shows the alignment of the ALS genotype-specific down-regulated proteins. Enrichment analysis shows the top KEGG pathways and Gene Ontology (GO) terms of SOD1- (**b**), C9orf72- (**c**), FUS- (**d**) and TARDBP-specific down-regulated protein IDs (**e**).

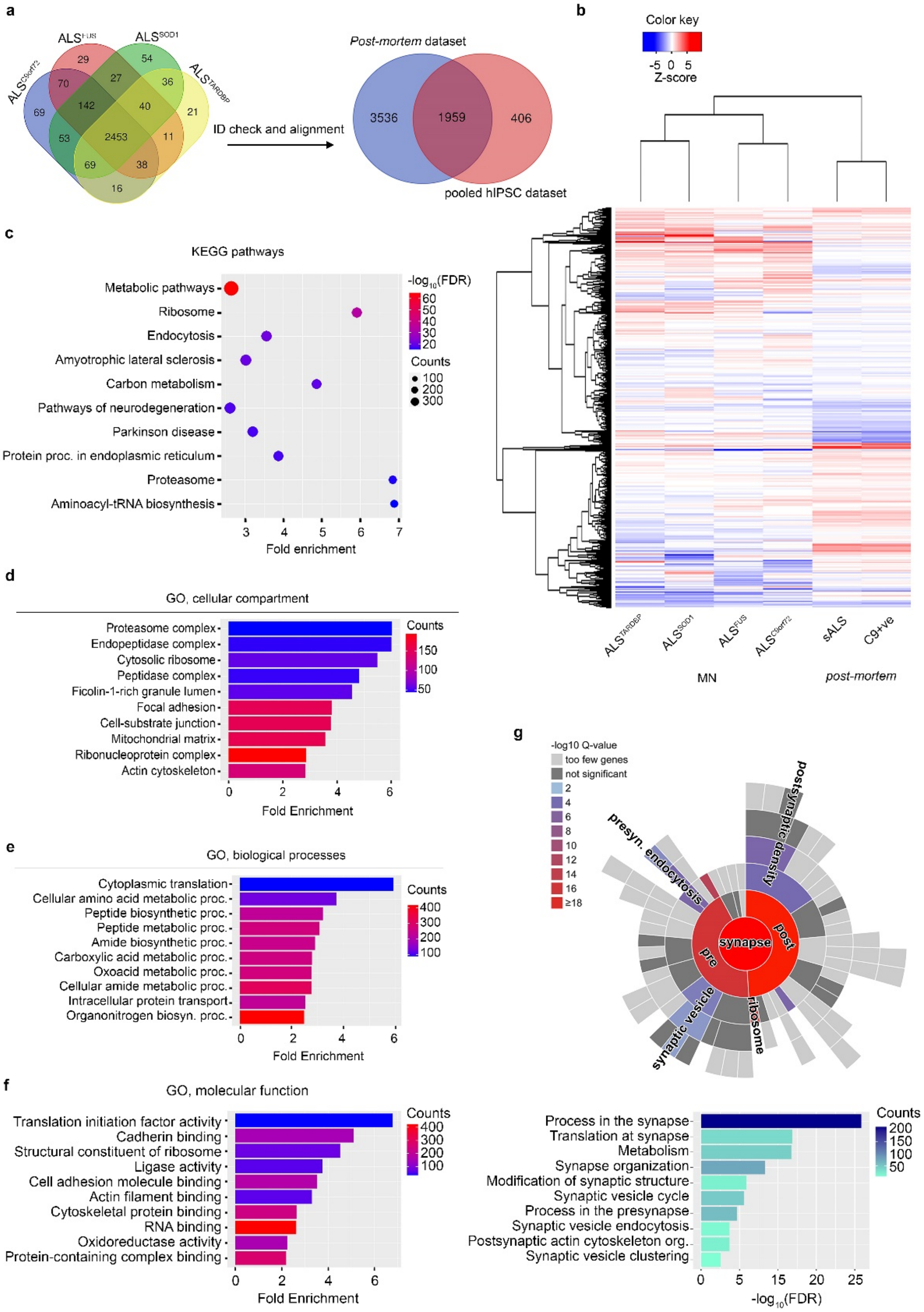
a
ALS^FUS
ALS^SOD1
ALS^C9orf72
ALS^TARDBP
29
54
70
27
36
69
142
40
21
53
2453
11
69
38
16
ID check and alignment
Post-mortem dataset
3536
1959
406
pooled hIPSC dataset
b
Color key
-5 0 5
Z-score
ALS^TARDBP
ALS^SOD1
ALS^FUS
ALS^C9orf72
sALS
C9+ve
MN
post-mortem
c
KEGG pathways
Metabolic pathways
Ribosome
Endocytosis
Amyotrophic lateral sclerosis
Carbon metabolism
Pathways of neurodegeneration
Parkinson disease
Protein proc. in endoplasmic reticulum
Proteasome
Aminoacyl-tRNA biosynthesis
3 4 5 6 7
Fold enrichment
-log10(FDR)
60
50
40
30
20
Counts
100
200
300
d
GO, cellular compartment
Proteasome complex
Endopeptidase complex
Cytosolic ribosome
Peptidase complex
Ficolin-1-rich granule lumen
Focal adhesion
Cell-substrate junction
Mitochondrial matrix
Ribonucleoprotein complex
Actin cytoskeleton
0 2 4 6
Fold Enrichment
Counts
150
100
50
e
GO, biological processes
Cytoplasmic translation
Cellular amino acid metabolic proc.
Peptide biosynthetic proc.
Peptide metabolic proc.
Amide biosynthetic proc.
Carboxylic acid metabolic proc.
Oxoacid metabolic proc.
Cellular amide metabolic proc.
Intracellular protein transport
Organonitrogen biosyn. proc.
0 2 4 6
Fold Enrichment
Counts
400
300
200
100
f
GO, molecular function
Translation initiation factor activity
Cadherin binding
Structural constituent of ribosome
Ligase activity
Cell adhesion molecule binding
Actin filament binding
Cytoskeletal protein binding
RNA binding
Oxidoreductase activity
Protein-containing complex binding
0 2 4 6
Fold Enrichment
Counts
400
300
200
100
g
-log10 Q-value
too few genes
not significant
2
4
6
8
10
12
14
16
≥18
postsynaptic density
presyn. endocytosis
synapse
post
pre
synaptic vesicle
ribosome
Process in the synapse
Translation at synapse
Metabolism
Synapse organization
Modification of synaptic structure
Synaptic vesicle cycle
Process in the presynapse
Synaptic vesicle endocytosis
Postsynaptic actin cytoskeleton org.
Synaptic vesicle clustering
0 5 15 20 25
-log10(FDR)
Counts
200
150
100
50

Supplementary Fig. 12 Investigation of the overlap between ALS hiPSC-derived MNs and post-mortem dataset. **a** Venn diagrams show the filtering and alignment of hIPSC lines and post-mortem dataset. After ID check using only Swiss-Prot protein IDs, 2453 congruent proteins were found between the different ALS cell lines. This dataset was aligned with the human post-mortem proteomics dataset revealing 1959 common proteins visualized by heat map (**b**). **c** Enrichment analysis shows the top 10 KEGG pathways based on fold enrichment, sorted by -log10(FDR) and gene counts (dots size). **d-f** Top 10 Gene Ontology (GO) terms demonstrate the most enriched functional and cellular clusters. **g** Subsynaptic analysis by SynGO database present the most enriched synaptic terms including synaptic vesicle cycle and endocytosis.

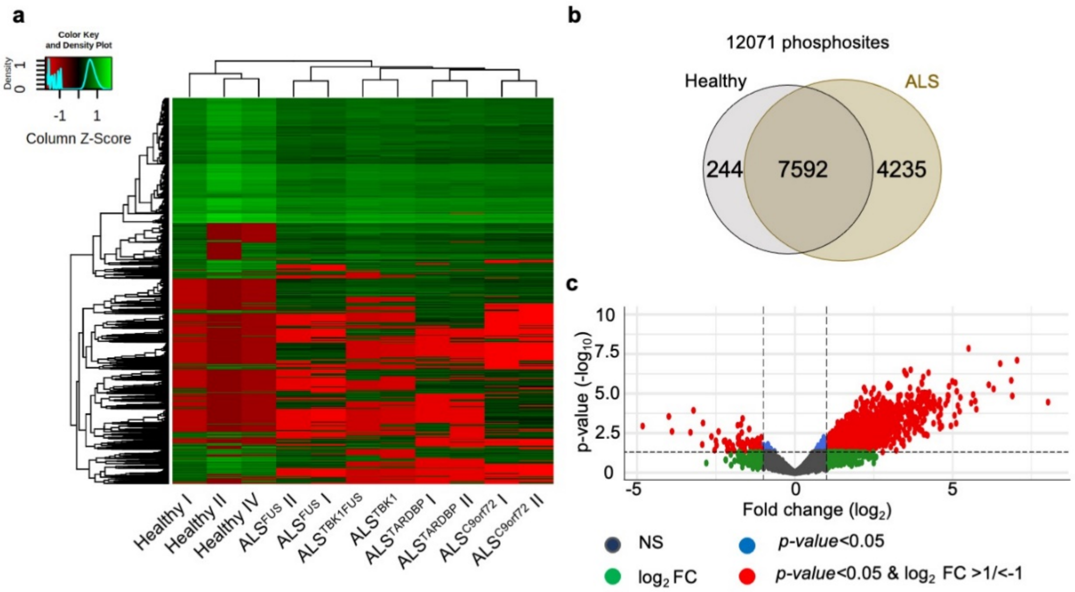

Supplementary Fig. 13 Total phosphoproteome analysis in hiPSC-derived MNs. **a** Heatmap based on all the unique phosphosites identified in human MN. Already at the level of total phosphoproteome, a separation between controls and ALS mutants is detectable. The 4 subgroups of patients cluster according to their mutated gene. **b** Venn diagram shows the distribution of the 12071 detected non-redundant phosphosites in both experimental groups. **c** Volcano plot represents the significantly donw- and up-regulated phosphosites in the ALS group

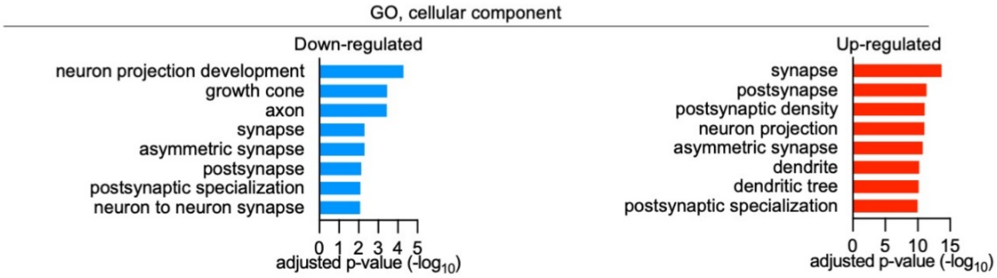

Supplementary Fig. 14 Most enriched cellular fractions in ALS phosphoproteome. Significantly down- and up-regulated GO (cellular component) terms in ALS based on the phosphoproteome dataset

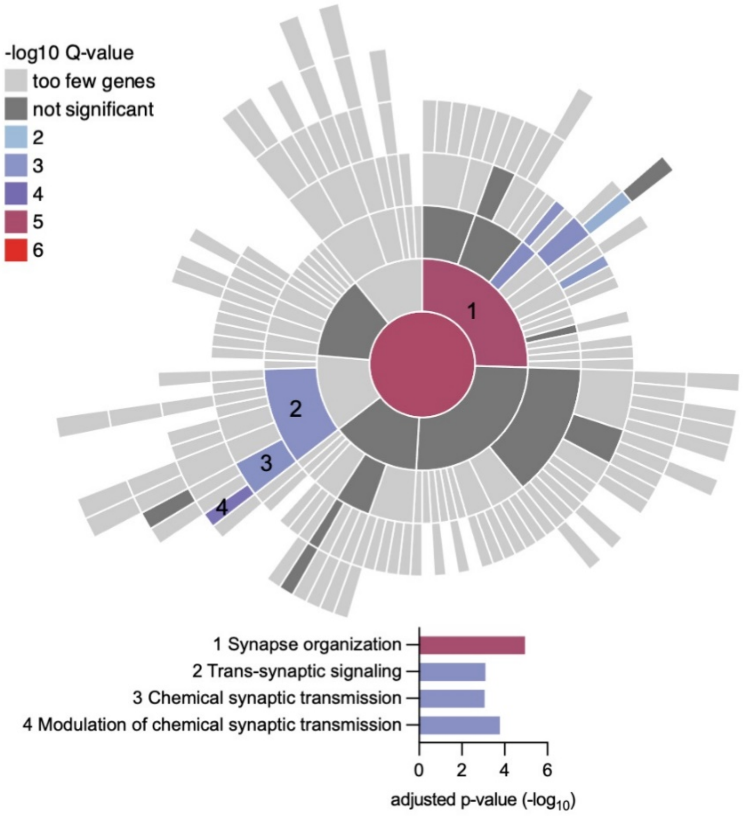

Supplementary Fig. 15 Phosphoproteome analysis confirms synaptic alterations in ALS. SynGO analysis based on the phosphoproteome dataset revealing a significant enrichment in pathways linked to synapse organization, signaling and transmission

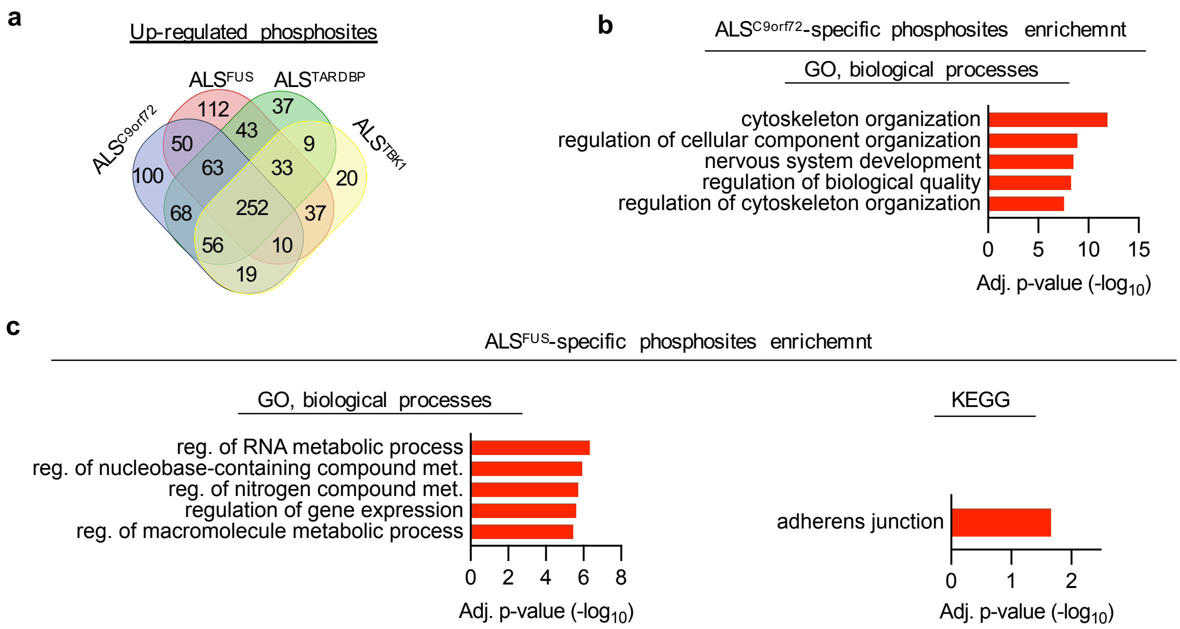

Supplementary Fig. 16 Alignment and enrichment analysis of the mutant cell line-specific up-regulated phosphosites. **a** Venn diagram shows the alignment of the ALS genotype-specific down-regulated proteins. Enrichment analysis shows the top KEGG pathways and Gene Ontology (GO) terms of C9orf72- (**b**) and FUS-mutant cultures (**c**).

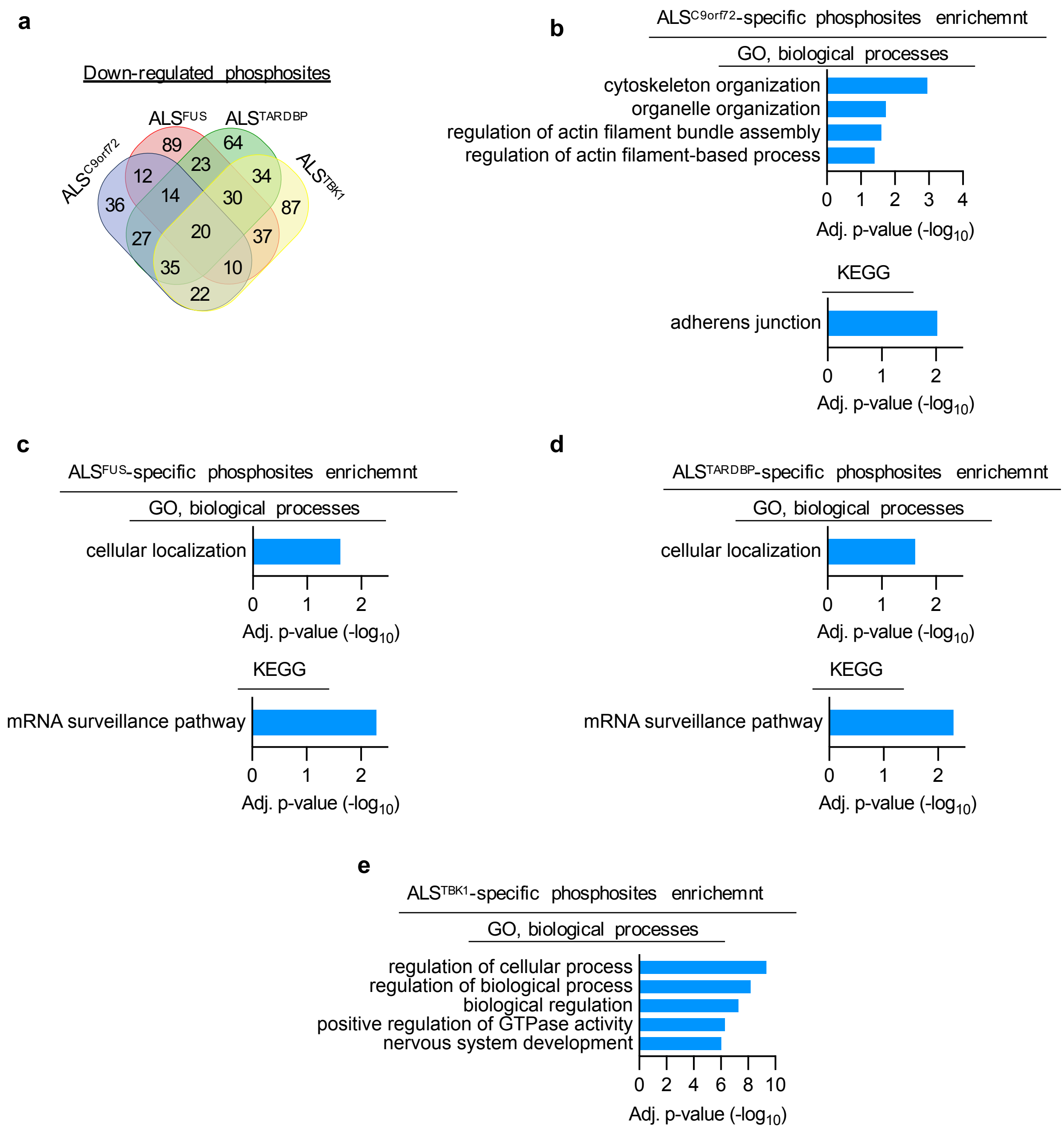

Supplementary Fig. 17 Alignment and enrichment analysis of the mutant cell line-specific down-regulated phosphosites. **a** Venn diagram shows the alignment of the ALS genotype-specific down-regulated proteins. Enrichment analysis shows the top KEGG pathways and Gene Ontology (GO) terms of C9orf72- (**b**), FUS- (**c**), TARDBP- (**d**) and TBK1-mutant cultures (**e**).

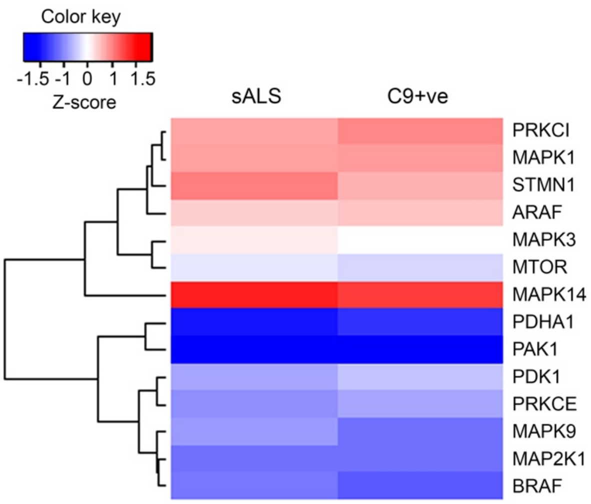

Supplementary Fig. 18 Altered kinase levels in the ALS synaptoneurosomes. The kinases predicted to be responsible of the altered phosphoproteome in hiPSC-derived ALS MNs are altered in the *post-mortem* synaptic fraction of sALS and C9+ve samples.

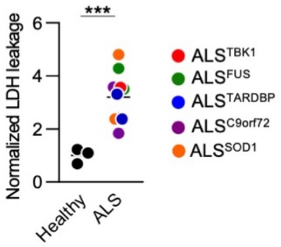

Supplementary Fig. 19 Increased cell stress and apoptosis in ALS MNs. Cultures of mutant MNs show significantly higher levels of leaked LDH at DIV 42 in comparison to healthy controls. Welch's *t*-test ***$P < 0.001$. $n = 3$ independent cultures

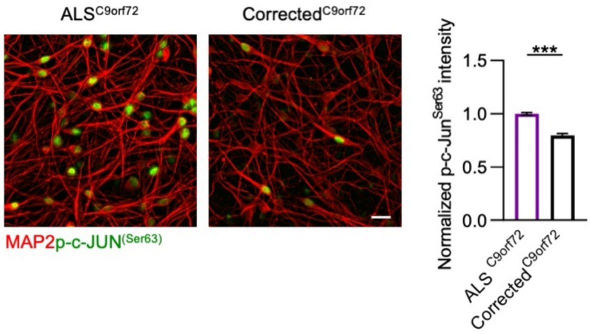

Supplementary Fig. 20 Decreased phosphorylation levels of phospho-cJun^{Ser63} in corrected C9orf72-mutant MNs. Correcting the pathogenic *GGGGCC* expansion in a C9orf72-mutant line using CRISPR-Cas9 significantly reduces phospho-cJun^{Ser63} intensity compared to C9orf72-mutant MN. Graph shows mean±SEM. Data analysed by Mann-Whitney test ***$P < 0.001$. $n = 3$ independent cultures. Scale bar: 25 μm

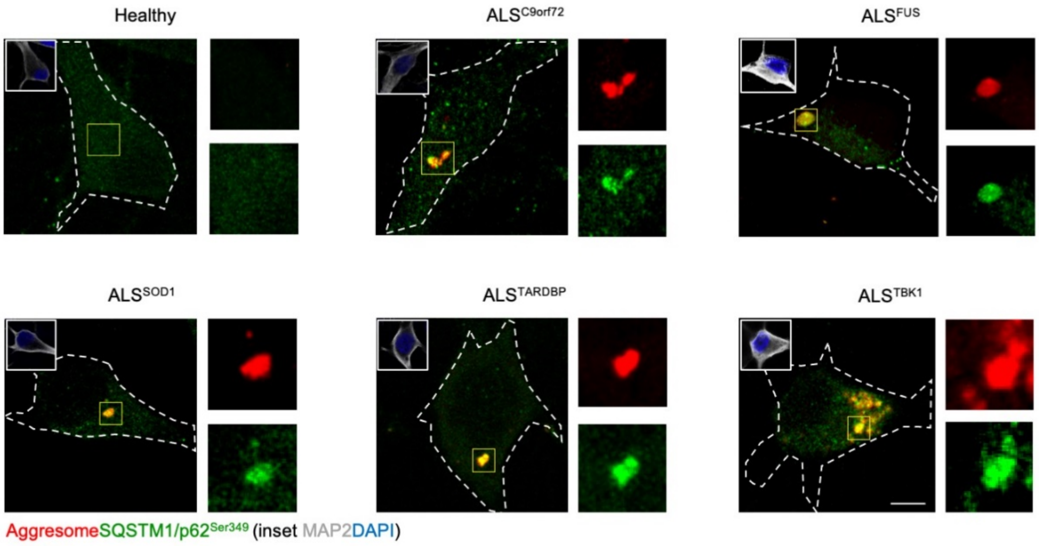

Supplementary Fig. 21 Aggregate accumulation in hiPSC-derived ALS MNs. Representative confocal images showing colocalization of phospho-SQSTM1/p62^{Ser349} within cytotoxic aggresomes in the presence of pathogenic mutations. Scale bar: 5 µm. Dashed line represents the cell soma

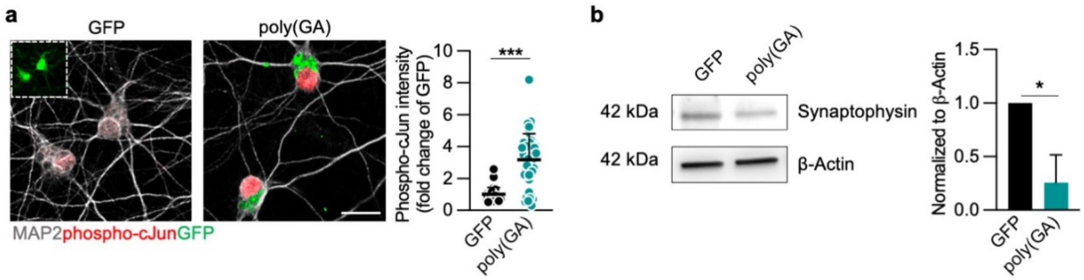

Supplementary Fig. 22 Overexpression of poly(GA) in primary neurons recapitulates alterations similar to those observed in human ALS MNs. **a** Overexpression of poly(GA) significantly increases the levels of nuclear phospho-cJun^{Ser63} compared to EGFP-positive cells. Mann-Whitney test ***$P < 0.001$. n= 3 independent cultures. Scale bar:20 µm. **b** Poly(GA)+ cortical neurons have significantly lower levels of SYP in comparison to EGFP-expressing neurons. Graph represents mean±SEM and data were analyzed by Mann-Whitney test *$P < 0.05$. n= 3 independent cultures

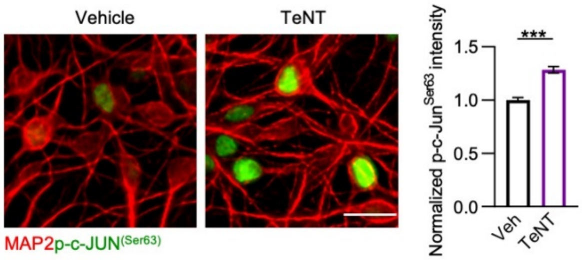

Supplementary Fig. 23 Pharmacological inhibition of synaptic vesicle release increases phospho-cJun^{Ser63} levels in Healthy cultures. Inhibition of synaptic vesicle release after 24 hours of treatment with tetanus neurotoxin (TeNT) significantly increases the levels of phospho-cJun^{Ser63} in Healthy MNs compared to vehicle-treated ones. Graph show mean±SEM analysed by Mann-Whitney test ***$P < 0.001$. $n = 3$ independent treatments performed with the Healthy I line. Scale bar: 25 μm

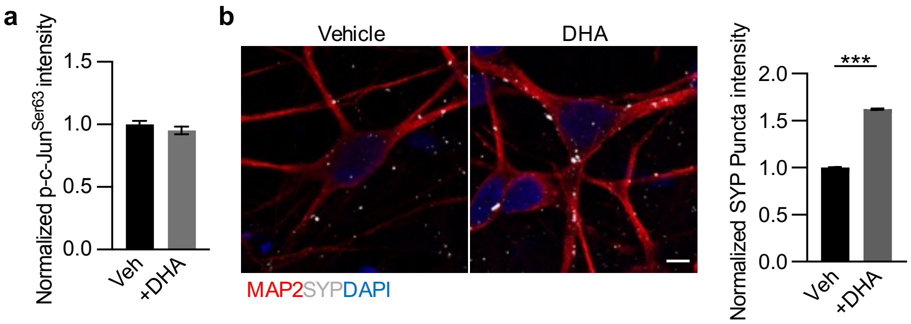

Supplementary Fig. 24 DHA treatment in Healthy hiPSC-derived MNs cultures. **a** DHA treatment does not modify the level of p phospho-cJun^{Ser63} in control cultures. **b** Treatment of control MN with DHA significantly increases SYP levels in comparison to vehicle-treated cultures. Graph shows mean±SEM. Mann-Whitney test ***$P < 0.001$. n= 3 independent treatments performed with the Healthy I line. Scale bar: 5 µm

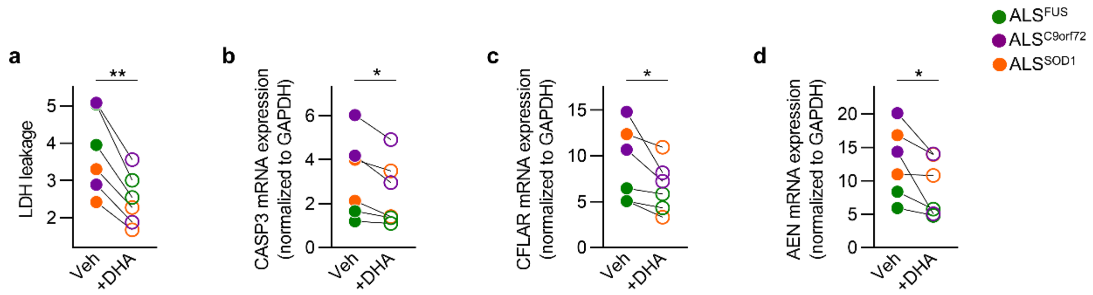

Supplementary Fig. 25 DHA treatment reduces apoptosis in ALS MNs. **a** Treatment with DHA exerts a beneficial effect in mutant cultures by significantly decreasing the levels of leaked LDH in comparison to vehicle-treated cultures. Paired t-test $^{**}P < 0.01$. $n = 3$ independent treatments with each hiPSC line. **b-d** Single tube qPCR experiments showing the down-regulation of JUN apoptotic targets *CASP3*, *CFLAR* and *AEN* in ALS MNs after DHA treatment. Paired t-test $^{*}P < 0.05$. $n = 3$ independent treatments with each hiPSC line.
